# Artificial Intelligence Learning Semantics via External Resources for Classifying Diagnosis Codes in Discharge Notes

**DOI:** 10.2196/jmir.8344

**Published:** 2017-11-06

**Authors:** Chin Lin, Chia-Jung Hsu, Yu-Sheng Lou, Shih-Jen Yeh, Chia-Cheng Lee, Sui-Lung Su, Hsiang-Cheng Chen

**Affiliations:** ^1^ School of Public Health National Defense Medical Center Taipei Taiwan; ^2^ Department of Research and Development National Defense Medical Center Taipei Taiwan; ^3^ Planning and Management Office Tri-Service General Hospital National Defense Medical Center Taipei Taiwan; ^4^ Da-Yeh University Changhua Taiwan; ^5^ Division of Rheumatology/Immunology/Allergy, Department of Internal Medicine Tri-Service General Hospital National Defense Medical Center Taipei Taiwan

**Keywords:** word embedding, convolutional neural network, neural networks (computer), natural language processing, text mining, data mining, machine learning, electronic medical records, electronic health records

## Abstract

**Background:**

Automated disease code classification using free-text medical information is important for public health surveillance. However, traditional natural language processing (NLP) pipelines are limited, so we propose a method combining word embedding with a convolutional neural network (CNN).

**Objective:**

Our objective was to compare the performance of traditional pipelines (NLP plus supervised machine learning models) with that of word embedding combined with a CNN in conducting a classification task identifying *International Classification of Diseases, Tenth Revision, Clinical Modification* (*ICD-10-CM*) diagnosis codes in discharge notes.

**Methods:**

We used 2 classification methods: (1) extracting from discharge notes some features (terms, n-gram phrases, and SNOMED CT categories) that we used to train a set of supervised machine learning models (support vector machine, random forests, and gradient boosting machine), and (2) building a feature matrix, by a pretrained word embedding model, that we used to train a CNN. We used these methods to identify the chapter-level *ICD-10-CM* diagnosis codes in a set of discharge notes. We conducted the evaluation using 103,390 discharge notes covering patients hospitalized from June 1, 2015 to January 31, 2017 in the Tri-Service General Hospital in Taipei, Taiwan. We used the receiver operating characteristic curve as an evaluation measure, and calculated the area under the curve (AUC) and F-measure as the global measure of effectiveness.

**Results:**

In 5-fold cross-validation tests, our method had a higher testing accuracy (mean AUC 0.9696; mean F-measure 0.9086) than traditional NLP-based approaches (mean AUC range 0.8183-0.9571; mean F-measure range 0.5050-0.8739). A real-world simulation that split the training sample and the testing sample by date verified this result (mean AUC 0.9645; mean F-measure 0.9003 using the proposed method). Further analysis showed that the convolutional layers of the CNN effectively identified a large number of keywords and automatically extracted enough concepts to predict the diagnosis codes.

**Conclusions:**

Word embedding combined with a CNN showed outstanding performance compared with traditional methods, needing very little data preprocessing. This shows that future studies will not be limited by incomplete dictionaries. A large amount of unstructured information from free-text medical writing will be extracted by automated approaches in the future, and we believe that the health care field is about to enter the age of big data.

## Introduction

Public health surveillance systems are important for identifying unusual events of public health importance and will provide information for public health action [1]. However, most surveillance systems can only use structured data, such as *International Classification of Diseases, Tenth Revision, Clinical Modification* (*ICD-10-CM*) diagnosis codes. The current methods for collecting this structured information usually involve manual identification, but manual identification of disease codes from free-text clinical narratives is laborious and costly. Moreover, most surveillance systems do not have enough expert clinical coders for real-time surveillance, and this leads to delays in the release of disease statistics. Government health administrators need timely information to rapidly assess disease prevention and health protection priorities. A timely and computer-based disease classification approach is required to further assist public health action.

Automated surveillance methods are increasingly being researched because of the increasing volume and accessibility of electronic medical data, and a range of studies have proven the feasibility of extracting structured information from clinical narratives [2-6]. Previous studies suggested that these text mining approaches would need to effectively deal with the idiosyncrasies of the clinical sublanguage to further improve performance [7]. However, compiling a complete medical dictionary may be impossible because of the variability of clinical vocabularies. Moreover, traditional natural language processing (NLP) pipelines can deal with synonyms but not similar terms, so supervised machine learning models often face the curse of dimensionality. For example, if we only want to identify infectious disease-related medical documents, bacteria names such as *Streptococcus pneumoniae* and *Mycobacterium tuberculosis* can actually be treated as similar for classification purposes. An effective text preprocessing approach would need to learn how to combine similar concepts, and current NLP pipelines often cannot deal with this issue.

Another important challenge for automated surveillance algorithms is emerging disease. For example, influenza H1N1 broke out in 2009 and could not have been recorded in any medical records before 2008. Traditional automatic methods based on term vectors cannot use new terms [2-6]. This weakness means that traditional methods cannot possibly implement a fully automated pipeline. The key reason that human experts can successfully identify emerging diseases is that humans can learn semantics from external resources. Traditionally, these external resources usually take the form of a dictionary, and this is what will be used in the NLP pipeline. However, dictionary construction is laborious, and it is still difficult to completely include all semantic relationships. In summary, traditional NLP pipelines are complex and inefficient, and successful automated surveillance methods will also need to include automatic handling of semantics.

Word embedding is a feature learning technique where vocabularies are mapped to vectors of real numbers [8,9]. Word2vec [10] and GloVe [11] are the 2 most popular word embedding algorithms. These methods showcase interesting linear substructures in the word vector space: word vectors for similar concepts are likewise close in terms of cosine similarity and Euclidean distance. This property may help us identify concept groups and reduce the data dimensionality in future machine learning algorithms. However, clinical narratives will be transformed into a matrix, and standardization to vectors with different length is difficult for general machine learning models. Convolutional neural networks (CNNs) use layers with convolving filters that are applied to local features, and they can handle matrix input [12]. CNNs were originally invented for computer vision applications and have subsequently been shown to achieve excellent results in semantic parsing [13], search query retrieval [14], and sentence classification [15]. The key reason for the success of CNNs is their fuzzy matching using convolving filters, and we believe that convolving filters are a great way to process similar texts involving the same concepts. A lot of words and phrases that are conceptually similar can be combined in a convolving filter via fuzzy matching technology, thereby reducing the data dimensionality and avoiding overfitting.

This project aimed to compare traditional machine learning pipelines (NLP plus supervised machine learning models) versus word embedding combined with a CNN in order to identify chapter-level *ICD-10-CM* diagnosis codes in discharge notes. We hoped to develop an efficient and effective real-time surveillance pipeline for disease statistics. In addition, we further analyzed the convolving filters of the CNN to understand their functions.

## Methods

### Data Source

The Tri-Service General Hospital, Taipei, Taiwan, supplied deidentified free-text discharge notes from June 1, 2015 to January 31, 2017. Research ethics approval was given by the institutional ethical committee and medical records office of the Tri-Service General Hospital to collect data without individual consent for sites where data are directly collected. The Tri-Service General Hospital is located in the Neihu District of Taipei under the name of National Defense Medical Center and provides medical service for service members, their family dependents, and civilians. It has been rated by the Ministry of Health and Welfare in Taiwan as a first-rate teaching hospital on the level of a medical center. The hospital has about 1700 beds and 6000 inpatients per month, and most inpatients are civilians. We collected a total of 103,390 discharge notes, and corrected misspellings using the Hunspell version 2.3 package [16] and a dictionary built using English Wikipedia and Gigaword [17]. *ICD-10-CM* codes had been used to label these discharge notes for the purpose of requesting health insurance fees, and the medical records department was responsible for their correctness. The Taiwan National Health Insurance Administration routinely samples a certain number of discharge notes for verification, and a wrongly labeled discharge note is punishable by a 10- to 20-fold fine. Discharge notes are often labeled with multiple *ICD-10-CM* codes, and all *ICD-10-CM* codes were truncated at the 1-character level. There are a total of 21 categories in the 2017 version. Table 1 shows the frequency distribution of 1-character-level codes. Neoplasms and diseases of the circulatory system were the most common *ICD-10-CM* codes in our hospital.

We used 2 testing procedures to assess the performance of the model. First, we conducted a 5-fold cross-validation test. Second, we created training and testing sets by splitting the sample by date (July 1, 2016), because this is more realistic. A classifier can only be trained using retrospective data in the real world, and it will be used to classify future data; the second testing process replicates this. All calculations were conducted on a Fujitsu RX2540M1 48-core CPU, 768 GB RAM server (Fujitsu Ltd, Tokyo, Japan), and the all-flash array was AccelStor NeoSapphire NS3505 (AccelStor, Inc, Taipei City, Taiwan) with a 5 TB serial advanced technology attachment-interface solid-state drive and connectivity of 56 GB/second FDR InfiniBand Quad Small Form-factor Pluggable (Fiberon Technologies, Inc, Westborough, MA, USA).

**Table 1:** Prevalence of different *International Classification of Diseases, Tenth Revision, Clinical Modification* (*ICD-10-CM*) chapter-level codes in discharge notes from the Tri-Service General Hospital, Taipei, Taiwan.

*ICD-10-CM* code	Definition	Stage of the study		
		Before June 30, 2016 (n=64,023) n (%)	After July 1, 2016 (n=39,367) n (%)	Full study period (n=103,390) n (%)
A00-B99	Certain infectious and parasitic diseases	7731 (12.1%)	5455 (13.9%)	13,186 (12.8%)
C00-D49	Neoplasms	20,585 (32.2%)	13,993 (35.5%)	34,578 (33.5%)
D50-D89	Diseases of the blood and blood-forming organs and certain disorders involving the immune mechanism	4516 (7.1%)	3132 (8.0%)	7648 (7.4%)
E00-E89	Endocrine, nutritional, and metabolic diseases	13,223 (20.7%)	8765 (22.3%)	21,988 (21.3%)
F01-F99	Mental, behavioral, and neurodevelopmental disorders	4612 (7.2%)	2942 (7.5%)	7554 (7.3%)
G00-G99	Diseases of the nervous system	3703 (5.8%)	2602 (6.6%)	6305 (6.1%)
H00-H59	Diseases of the eye and adnexa	2337 (3.7%)	1374 (3.5%)	3711 (3.6%)
H60-H95	Diseases of the ear and mastoid process	802 (1.3%)	470 (1.2%)	1272 (1.2%)
I00-I99	Diseases of the circulatory system	17,650 (27.6%)	11,465 (29.1%)	29,115 (28.2%)
J00-J99	Diseases of the respiratory system	7743 (12.1%)	5584 (14.2%)	13,327 (13.0%)
K00-K95	Diseases of the digestive system	12,849 (20.1%)	8444 (21.4%)	21,293 (20.6%)
L00-L99	Diseases of the skin and subcutaneous tissue	2568 (4.0%)	1711 (4.3%)	4279 (4.1%)
M00-M99	Diseases of the musculoskeletal system and connective tissue	9170 (14.3%)	5152 (13.1%)	14,322 (13.9%)
N00-N99	Diseases of the genitourinary system	9929 (15.5%)	7325 (18.6%)	17,254 (16.8%)
O00-O9A	Pregnancy, childbirth, and the puerperium	2509 (3.9%)	1271 (3.2%)	3780 (3.7%)
P00-P96	Certain conditions originating in the perinatal period	793 (1.2%)	493 (1.3%)	1286 (1.2%)
Q00-Q99	Congenital malformations, deformations, and chromosomal abnormalities	927 (1.4%)	513 (1.3%)	1440 (1.4%)
R00-R99	Symptoms, signs, and abnormal clinical and laboratory findings, not elsewhere classified	5271 (8.2%)	3824 (9.7%)	9095 (8.9%)
S00-T88	Injury, poisoning, and certain other consequences of external causes	6272 (9.8%)	4564 (11.6%)	10,836 (10.6%)
V00-Y99	External causes of morbidity	791 (1.2%)	68 (0.2%)	859 (0.8%)
Z00-Z99	Factors influencing health status and contact with health services	15,488 (24.2%)	10,093 (25.6%)	25,581 (24.8%)

### Traditional Free-Text Classification Techniques

Traditional classification techniques often combine an NLP pipeline and a classifier to conduct free-text medical writing classification tasks. We extracted the detailed features from the discharge notes by the NLP pipeline; then *ICD-10-CM* codes were assigned by human experts to each discharge note. We used the labeled features to train a classifier, and we used the well-trained model to predict the unlabeled testing data.

In this study, we used a 2-part NLP pipeline to extract the discharge note features. First, word-based features were directly extracted from the free-text description and n-gram phrases (n range 2-5) were generated by the RWeka version 0.4-30 package [18]. To reduce the complexity of the data, we only included n-gram phrases with counts >10. Second, we used SNOMED CT International Edition version 20170131 categories to integrate synonyms. We used the bag-of-words model to vectorize the extracted features (1 vector per discharge note) and transformed these feature vectors into a document-term matrix using the tm version 0.7 package [19]. This matrix was then the input into the following machine learning models.

#### Support Vector Machine

Support vector machines (SVMs) are common classifiers in the machine learning field. They map all samples onto a hyperplane and divide them by a clear gap. In addition, kernel tricks are used to extend this hyperplane. SVMs are proven to have the best performance in free-text medical writing classification, compared with naive Bayes classifiers, C4.5 decision trees, and adaptive boosting [20]. In this study, we used the 4 most common kernel tricks: linear, polynomial (degree=3), radial basis, and sigmoid. We used the e1071 package (R package version 1.6-8) [21] as the SVM implementation and set all other parameters to their default values.

#### Random Forest

Random forests (RFs) construct multiple decision trees and use information from each tree to make predictions. It was the best-performing classification model in a previous text classification study [22], compared with SVMs, naive Bayes classifiers, and the k-nearest neighbors algorithm. We used the H2O version 3.10.2.2 package [23] as the RF implementation and set all parameters to their default values.

#### Gradient Boosting Machine

Gradient boosting machines (GBMs) are also ensembles of weak decision trees, where the gradient boosting method is used to improve the predictive ability of each tree [24]. They use greedy function approximation to build a series of weak trees [25]. The H2O package also provides the function for the GBM implementation, and we set all parameters to their default values.

Using the “no free lunch” theorem [26], we combined a traditional NLP pipeline with the 3 abovementioned models and tested their performance on our task.

### Word Embedding Combined With a Convolutional Neural Network

Traditional NLP pipelines are limited by their preexisting dictionary and need to build a complex processing flow. Herein, we propose a method combining a word embedding model and a CNN. Word embedding technology is useful for integrating synonyms, and we used a pretrained GloVe model (English Wikipedia plus Gigaword) to vectorize the words. We selected a 50-dimensional model with 400,000 words because of computing time constraints. However, we believe that this was sufficient because there were only 19,064 words in our 103,390 discharge notes. We transformed each discharge note into an n×50 matrix for subsequent classification (where n is the number of words in the discharge note) and trained a CNN using these labeled matrixes.

**Figure 1:**
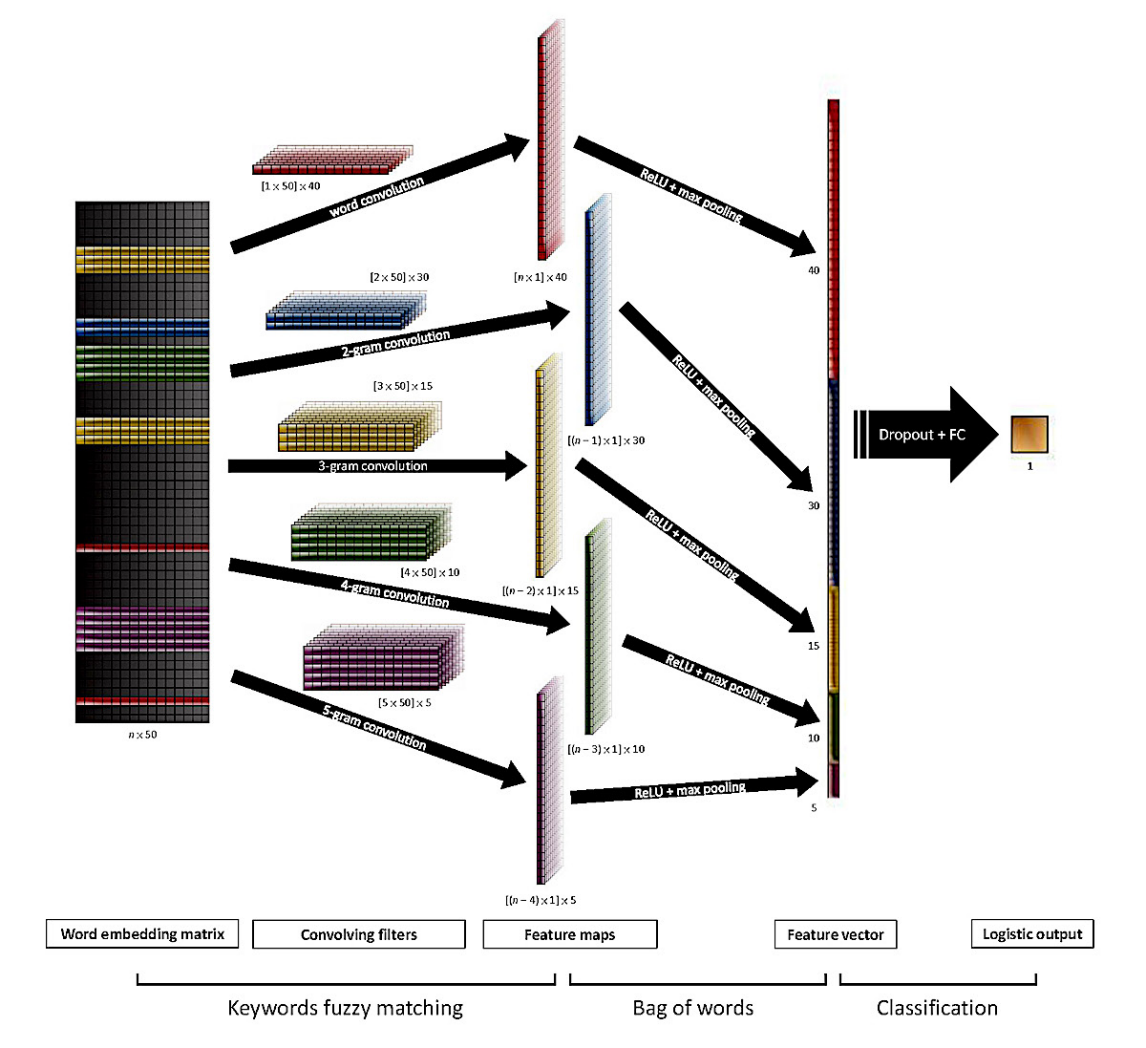
Model architecture with 5 convolution channels and 1 full connection (FC) layer. ReLU: rectified linear unit.

Although CNNs with various structures have been developed, we focused on a 1-layer CNN with a filter region size of 1-5 (corresponding to 1-5 n-gram phrases) to increase comparability with traditional machine learning technologies. In fact, these simple models have recently achieved remarkably strong performance [15,27,28]. Figure 1 shows the proposed model’s architecture. We set 5 convolution channels, and their convolution layers were as follows: (1) 40 convolving filters with a 1×50 region size, to identify the important words; (2) 30 convolving filters with a 2×50 region size, to identify the important 2-gram phrases; (3) 15 convolving filters with a 3×50 region size, to identify the important 3-gram phrases; (4) 10 convolving filters with a 4×50 region size, to identify the important 4-gram phrases; and (5) 5 convolving filters with a 5×50 region size, to identify the important 5-gram phrases. These convolution layers were connected to a rectified linear unit layer to enhance the nonlinearity of the network. We then applied a max pooling layer over the feature map and took the maximum value. The above steps are similar to those of the keyword recognition process, and 100 features were extracted from each discharge note. To avoid the risk of overfitting, we used a dropout layer with a 50% drop rate after the convolution channels [29]. Finally, we used logistic regression to connect the features, and the cross-entropy loss function in the loss layer to train the CNN.

We used the MXNet version 0.8.0 package [30] to implement the above architecture. The settings used for the training model were as follows: (1) minibatch gradient descent with 1000 bench size for optimization; (2) learning rate=.05; (3) momentum coefficient=.9; (4) L2 regularization coefficient=.00001; and (5) tolerance of early stopping per 100 iterations=.0001. Multimedia Appendix 1 shows an example code for implementing the word embedding and CNNs for free-text discharge note classification.

### Model Details and Evaluation Index

We conducted oversampling processing for sufficiently regarding positive cases but not skewing by an overwhelming number of negative cases [31,32]. All the models return a continuous value to evaluate model performance. SVM-related models provide the decision values of the binary classifier; RF and GBM models provide the mean of the probabilities from the decision trees; and CNNs provide the probabilities calculated by the logistic function. We used the receiver operating characteristic curve as an evaluation measure, and the area under the curve (AUC) provided a global measure of effectiveness. Moreover, we provide the F-measure, which is calculated by following equations: *precision* = *TruePositives* / (*TruePositives* + *FalsePositives*); *recall* = *TruePositives* / (*TruePositives* + *FalseNegatives*); *F-measure* = (2 × *precision* × *recall*) / (*precision* + *recall*).

## Results

### Cross-Validation Test

Table 2 shows the global and lowest 5 means of the training and testing AUCs in the 5-fold cross-validation test. The proposed word embedding plus CNN method provided the highest AUCs (mean testing AUC = 0.9696; mean of the lowest 5 AUCs = 0.9135) and highest F-measures (mean testing F-measure = 0.9086; mean of the lowest 5 F-measures = 0.7651). It is worth noting that the SVM with the linear kernel trick had the highest mean testing AUC of all the traditional methods (mean testing AUC = 0.9571; mean of the lowest 5 AUCs = 0.8891). The performances of the RF, GBM, and linear SVM models were similar (mean testing AUCs of 0.9570, 0.9544, and 0.9571, respectively). However, the RF and GBM models were very inefficient in some tasks (as Multimedia Appendix 2 shows). The RF and GBM models had a lower mean testing AUC owing to the V00-Y99 *ICD-10-CM* code identification tasks; therefore, the linear SVM was a relatively stable model.

**Table 2:** Global (and lowest 5) means of training and testing AUCs^a^ in the 5-fold cross-validation test.

Pipeline		Training set		Testing set	
		AUC^b^	F-measure	AUC^b^	F-measure
**Traditional**					
	NLP^c^ + SVM^d^ (linear)	0.9947 (0.9836)	0.9546 (0.8560)	0.9571 (0.8891)	0.8606 (0.6387)
	NLP + SVM (polynomial)	0.8627 (0.6736)	0.5630 (0.2498)	0.8183 (0.6332)	0.5050 (0.2023)
	NLP + SVM (radial basis)	0.9565 (0.9146)	0.7984 (0.6613)	0.9363 (0.8582)	0.7569 (0.5352)
	NLP + SVM (sigmoid)	0.9518 (0.9021)	0.7852 (0.6368)	0.9325 (0.8526)	0.7498 (0.5313)
	NLP + RF^e^	0.9999 (0.9995)^f^	0.9864 (0.9628)	0.9570 (0.8800)	0.8739 (0.6475)
	NLP + GBM^g^	0.9996 (0.9990)	0.9868 (0.9660)	0.9544 (0.8722)	0.8691 (0.6458)
**Proposed**					
	GloVe^h^ + CNN^i^	0.9964 (0.9890)	0.9837 (0.9588)	0.9696 (0.9135)^f^	0.9086 (0.7651)

^a^AUC: area under the curve, calculated using the receiver operating characteristic curve.

^b^The results are presented as the mean AUC or F-measure (mean of the lowest 5 AUCs or F-measures). Detailed AUCs and F-measures for each chapter-level *International Classification of Diseases, Tenth Revision, Clinical Modification* (*ICD-10-CM*) diagnosis code are shown in Multimedia Appendix 2.

^c^NLP: natural language processing for feature extraction (terms, n-gram phrases, and SNOMED CT categories).

^d^SVM: support vector machine.

^e^RF: random forest.

^f^The best method for a specific index.

^g^GBM: gradient boosting machine.

^h^GloVe: a 50-dimensional word embedding model, pretrained using English Wikipedia and Gigaword.

^i^CNN: convolutional neural network.

### Real-World Test

Table 3 shows the global and lowest 5 means of the training and testing AUCs in the real-world test, where the testing samples were split by date. The results of this test were similar to those of the cross-validation test. The testing AUC in the real-world test was lower than that in the cross-validation test, possibly because the heterogeneity between the training and testing samples was higher in the real-world test owing to there being many cyclical diseases. However, our proposed method still had the highest performance on the testing set (mean testing AUC = 0.9645; mean testing F-measure = 0.9003; mean of the lowest 5 AUCs = 0.8952; mean of the lowest 5 F-measures = 0.7204) and achieved the best results in almost all tasks. Multimedia Appendix 3 shows the detailed training and testing AUCs. The testing AUC of the proposed method is only obviously worse than that of traditional methods for the Q00-Q99 code identification tasks. In addition, the performances of all methods were bad for the V00-Y99 code identification tasks.

### Convolving Filter Analysis

**Figure 2:**
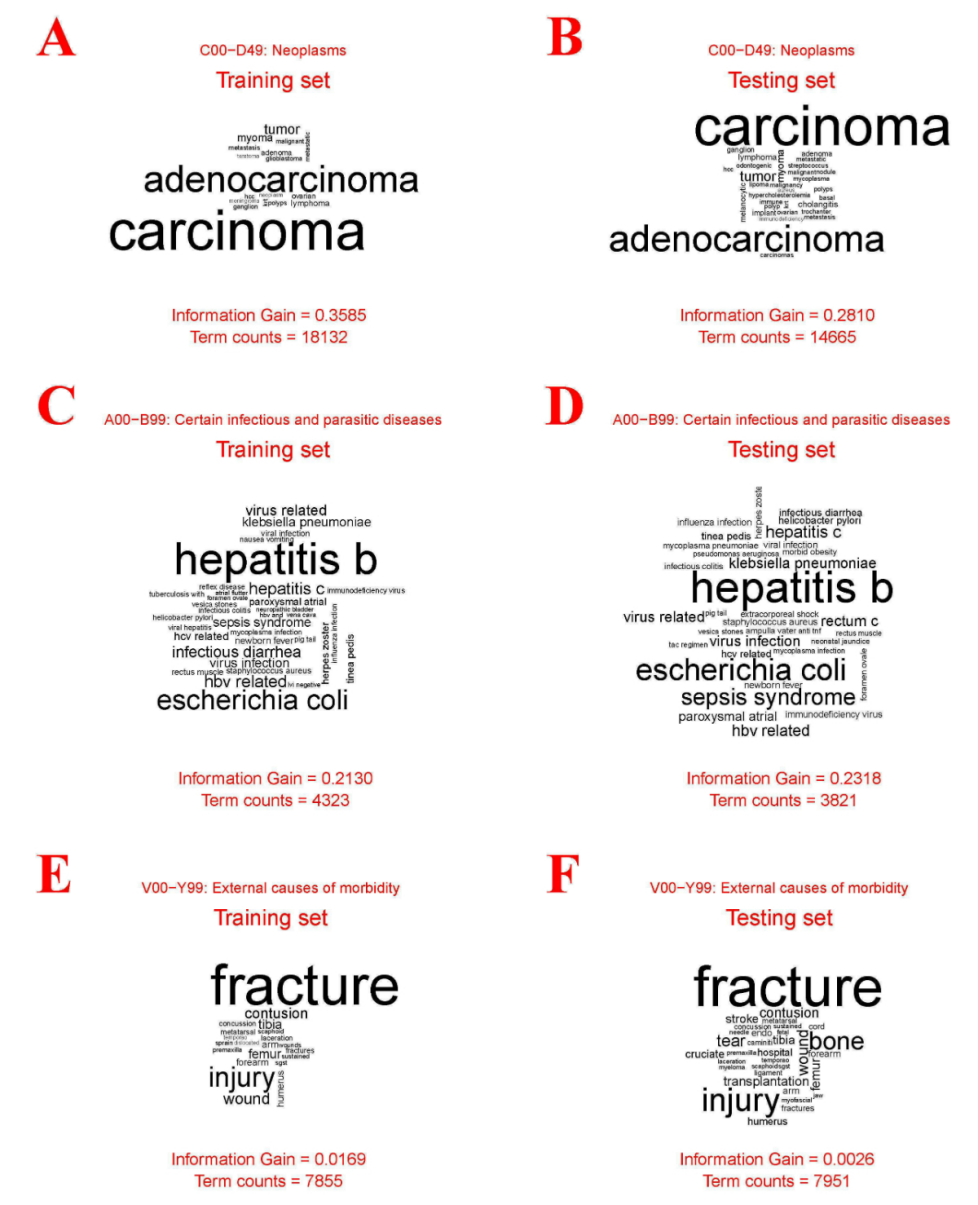
Visualization of selected convolving filters.

We visualized 3 of the convolving filters selected for the real-world test, as Figure 2 shows. Neoplasms were the most common *ICD-10-CM* codes in our hospital, and we selected the filter with highest information gain for these. Information gain can be estimated as IG(*C*, *F*) = H(*C*) − H(*C* | *F*), where *C* is the class (a specific *ICD-10-CM* code), *F* is the feature extracted by the convolving filter, and H is the information entropy function. This filter is a word filter that identified several cancer-related words, such as carcinoma and adenocarcinoma, when trained using the training data (Figure 2, panel A). As expected, these words, embodying similar concepts, were identified by the fuzzy matching technology. Moreover, the same words in the testing data were identified by this convolving filter (Figure 2, panel B). Figure 2, panel C shows a 2-gram convolving filter for certain infectious and parasitic diseases, which can identify many pathogens. It is worth mentioning that some pathogens absent in the training data were identified by this filter in the testing data (Figure 2, panel D). Identifying the external causes of morbidity was the most difficult task for all of the methods, and Figure 2, panel E shows the most important filter for this task. Some accident-related words were identified, such as fracture and injury, but these words were widely used in our discharge notes. The total number of discharge notes that included these words was 7855, but only 791 discharge notes were coded as V00-Y99 in the training set. This caused the information gain to be very low for the testing set (Figure 2, panel F).

**Figure 3:**
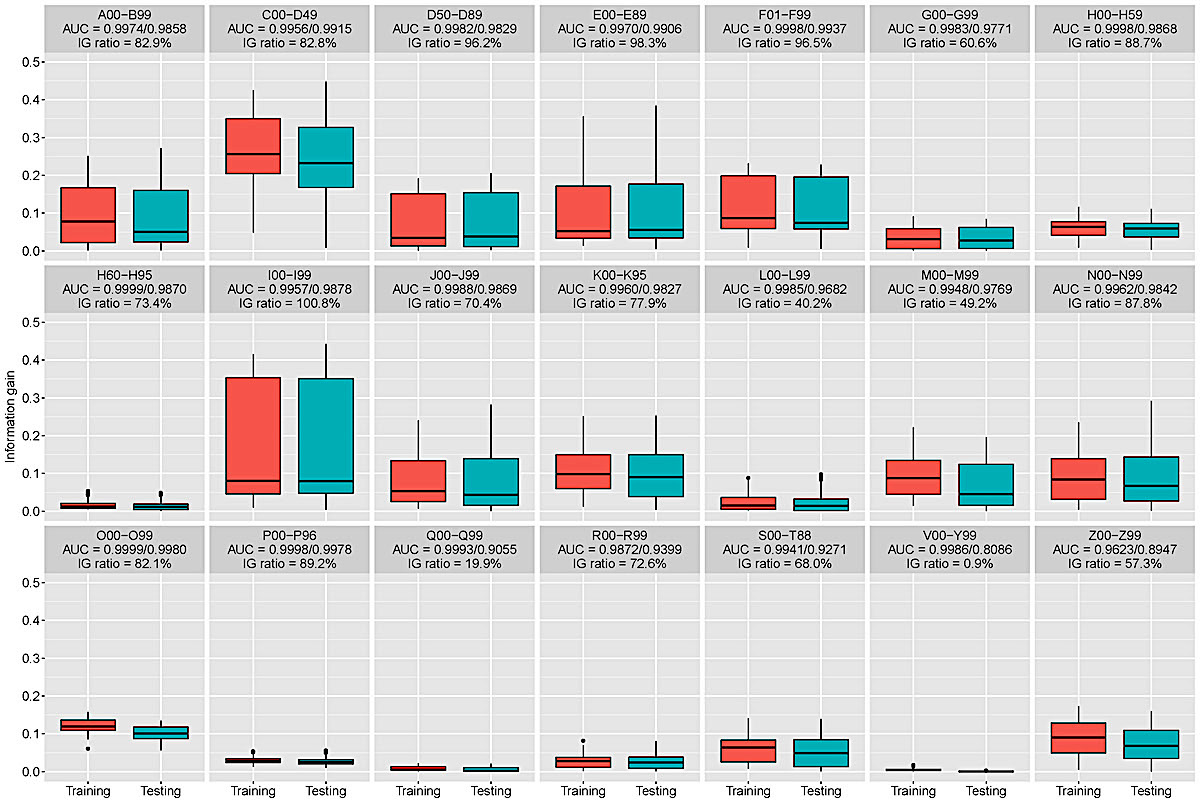
Information gains of the features extracted by the convolving filters in each classification task. AUC: area under the curve; IG: information gain.

Figure 3 shows the information gain distribution of the convolving filters in each task, demonstrating large differences between them. The highest-performing classification tasks often extracted high information gain features using convolving filters. Moreover, when the geometric mean of the information gain ratio between the training and testing sets was over 80%, the testing AUC was more than 0.98. It is worth noting that the information gain ratio was very low for Q00-Q99 and V00-Y99 (19.9% and 0.9%, respectively). This may explain the lower performance in these tasks.

**Table 3:** Global (and lowest 5) means of the training and testing AUCs^a^ in the real-world test.

Pipeline		Training set		Testing set	
		AUC^b^	F-measure	AUC^b^	F-measure
**Traditional**					
	NLP^c^ + SVM^d^ (linear)	0.9921 (0.9768)	0.9365 (0.7983)	0.9477 (0.8549)	0.8458 (0.5984)
	NLP + SVM (polynomial)	0.9103 (0.7975)	0.6316 (0.4045)	0.8716 (0.7400)	0.5761 (0.2802)
	NLP + SVM (radial basis)	0.9577 (0.9208)	0.7954 (0.6484)	0.9349 (0.8476)	0.7588 (0.5258)
	NLP + SVM (sigmoid)	0.9522 (0.9058)	0.7840 (0.6261)	0.9259 (0.8196)	0.7515 (0.5209)
	NLP + RF^e^	0.9996 (0.9985)^f^	0.9869 (0.9664)^f^	0.9483 (0.8484)	0.8582 (0.5901)
	NLP + GBM^g^	0.9995 (0.9985)	0.9821 (0.9562)	0.9462 (0.8416)	0.8568 (0.5948)
**Proposed**					
	GloVe^h^ + CNN^i^	0.9956 (0.9868)	0.9803 (0.9523)	0.9645 (0.8952)^f^	0.9003 (0.7204)^f^

^a^AUC: area under the curve, calculated using the receiver operating characteristic curve.

^b^The results are presented as the mean AUC or F-measure (mean of the lowest 5 AUCs or F-measures). Detailed AUCs and F-measures for each chapter-level *International Classification of Diseases, Tenth Revision, Clinical Modification* (*ICD-10-CM*) diagnosis code are shown in Multimedia Appendix 3.

^c^NLP: natural language processing for feature extraction (terms, n-gram phrases, and SNOMED CT categories).

^d^SVM: support vector machine.

^e^RF: random forest.

^f^The best method for a specific index.

^g^GBM: gradient boosting machine.

^h^GloVe: a 50-dimensional word embedding model, pretrained using English Wikipedia and Gigaword.

^i^CNN: convolutional neural network.

## Discussion

### Principal Findings

The proposed method, which combines word embedding with a CNN, had a higher testing accuracy than all traditional NLP-based approaches, regardless of the situation. Further analysis showed that convolving filters had fuzzy matching abilities, which greatly reduced the data dimension for the final classification task. Moreover, the training AUCs of the traditional methods were very close to 1. This means that there was no possibility of improvement, and the larger difference between training set and testing set performances implies overfitting.

Arbitrary free-text medical narratives include many word combinations, and there is no good way of integrating similar terms using the current NLP pipelines. Previous studies have highlighted this issue and suggested that improvements are possible by dealing more effectively with the idiosyncrasies of the clinical sublanguage [7]. We believe that our proposal has an advantage in this respect. The used fuzzy matching technology offers a real chance of reducing the risk of overfitting. This is not surprising, as CNNs have achieved excellent results in some text mining tasks [13-15,22,27,28]. This study also demonstrated the advantages of using CNNs for free-text medical narrative classification.

Our proposed method not only increased the accuracy compared with traditional methods, but also can avoid troublesome data preprocessing. Our solution for avoiding troublesome data preprocessing is based on word embedding, which can learn semantics from external resources. The vocabularies are mapped to vectors of real numbers, and the word vectors for similar concepts are likewise close. In our work, a discharge note is converted into an n×50 matrix, where n is the number of words, and CNN classifies this matrix based on our designed convolving filters. Because the word vectors for similar concepts are likewise close in terms, convolutional layers effectively identified a large number of keywords in a convolving filter (data shown in Figure 2.). Finally, we used the document features extracted by these convolving filters to identify *ICD-10-CM* diagnosis codes. This simple idea effectively deals with the idiosyncrasies of the clinical sublanguage, so the proposed method does not require data preprocessing by external dictionaries.

All the classifiers used in this study performed poorly on V00-Y99 (external causes of morbidity) coding tasks, which may be attributed to sparse testing data (0.2%). A previous study found that classifier performance was better on common cancers than on rare cancers [2]. However, the performance of the proposed method was clearly better than that of traditional methods. The Q00-Q99 (congenital malformations, deformations, and chromosomal abnormalities) coding tasks were the next key point, as our method was obviously worse than traditional methods in these tasks. After further analysis, we found that the most common second-level *ICD-10-CM* diagnosis codes in Q00-Q99 are Q80-Q89 (other congenital malformations), and the words used in these discharge notes were really complex. This means that our CNN may have needed more convolving filters to handle this issue. After we doubled the number of filters and retrained the CNN, the testing AUC greatly improved (testing AUCs of 0.9203 and 0.9235 in the cross-validation test and the real-world test, respectively). Hence, although a simple 1-layer CNN has already shown outstanding performance in our experiments, we believe that there are many opportunities to improve the performance of the proposed model.

All traditional term-based classifiers face the problem that emerging diseases cannot possibly be correctly classified. For example, influenza H1N1 could not possibly have been recorded in clinical narratives from 2000 to 2007, so term-based classifiers could not have been aware of the H1N1 pandemic of 2009 [3]. Our method can handle this problem using fuzzy matching technology. Although H1N1 was not recorded in discharge notes from 2000 to 2007, there was enough information to allow the machine to understand that H1N1 was an influenza subtype. In our pretrained GloVe model, H1N1 was very close to some influenza-related terms, such as “swine,” “influenza,” “flu,” and “H5N1” (the cosine similarities were 0.835, 0.832, 0.831, and 0.716, respectively). Thus, we believe that convolving filters could still have correctly identified H1N1 and classified related discharge notes as A00-B99 (certain infectious and parasitic diseases), but more precise coding would have been difficult. Thus, retraining or incrementally updating the classifiers would still be necessary; otherwise, emerging diseases would be merged into similar disease categories. However, this is still an important breakthrough in the free-text medical writing classification task.

Previous studies described the classification methods used by human experts, and several rule-based approaches have demonstrated superior performance [3,33]. The only problem with rule-based approaches is that adding new diseases requires the development of new models and rules. RF models use an ensemble of decision trees, where each interior node is differentiated on the basis of 1 of the terms. We consider the similarity between RF and rule-based approaches to be higher than with the proposed CNN. The machine must imitate human behavior patterns to improve its correctness. The RF model showed better performance than traditional classifiers in most identification tasks (mean testing ranks of 3.000 and 3.190 in the cross-validation test and real-world test, respectively), possibly attributed to the RF model having a similar identification process to that of human experts. The proposed CNN architecture uses a logistic function for output, similar to a linear SVM, although nonlinear SVMs showed a lower training AUC, which may have been due to wrong assumptions about the relationship between features and the outcome. This evidence shows that the assumption of a linear relationship between extracted features and outcome is better than a nonlinear assumption, and the architecture of our CNN also follows this linear assumption in its last layer. However, rule-based approaches are more inclined to use positive terms than negative ones [3,33], so the architecture of RF or GBM is better than a linear classifier. The proposed CNN showed the highest accuracy; the key to success is not our network architecture but the fuzzy term matching technology. Fuzzy term matching reduces the hazard of overfitting, and the mean training AUCs for the RF and GBM models were higher than those for the other models, possibly indicating that overfitting is more risky in RF and GBM models. In summary, we consider that a deeper CNN may provide more accurate predictive ability. Further studies need to consider this to improve the performance of word embedding combined with a CNN.

Outbreaks of deliberate and natural infectious disease can lead to massive casualties unless public health actions are promptly instituted [34]. Thus, many countries have been building real-time infectious disease surveillance systems, such as the Real-time Outbreak and Disease Surveillance system [35]. The implementation principle of the Real-time Outbreak and Disease Surveillance system is through the structured *ICD* code, and it needs real-time manual identification by emergency physicians. However, this system cannot be extended to all diseases because a lot of resources are required. In addition to infectious diseases, other chronic diseases also need to be surveilled in real time [36]. Government health administrators need timely information to rapidly assess disease prevention and health protection priorities. A timely automated disease classification algorithm is required. Our proposed method provides a viable pipeline for implementing a disease surveillance system of all diseases. It not only improves classification performance but also avoids the inherent limitations of traditional methods. Subsequent studies can use this algorithm to further develop fully automated disease surveillance systems.

### Limitations

Several potential limitations of this study should be acknowledged. First, we used only a 50-dimensional GloVe model to process our data, to reduce computing time. However, even a 50-dimensional model has better performance than traditional methods. Thus, we believe that this will not affect our result and that our proposal is a better solution for conducting free-text medical narrative coding tasks. Second, this study included discharge notes from only a single hospital, so we cannot confirm how well it would generalize to other data sources. Although this study only provided a feasibility assessment for extrapolation over time, we believe that it still demonstrated the superiority of our method. Third, this study conducted the classification task only in discharge notes. Discharge notes describe only the presence of the disease, but do not include negative statements. Our CNN architecture includes 3- to 5-gram phrase identifiers, but further studies are still needed to apply this approach to patient progress notes to prove its ability.

### Conclusion

Our study showed that combining CNNs with word embedding is a viable analysis pipeline for disease classification from free-text medical narratives. Moreover, it showed outstanding performance compared with traditional NLP employing machine learning classifiers and may avoid troublesome data preprocessing. More complex CNNs could be used to further improve predictive performance, and future studies will not be limited by incomplete dictionaries. Because our data were collected from a single center, further studies can implement this algorithm in other hospitals. We hope our experiment will lead to a range of studies toward developing more efficient automated classification approaches and that a large amount of unstructured information will be extracted from free-text medical writing. We have developed a Web app to demonstrate our work [37]. Public health surveillance systems would become more efficient, and government health administrators would be able to take timely and correct action for disease prevention and health protection. When previously unlabeled clinical records are labeled using such an automated approach, we can obtain more data-driven clues to help promote the progress of medicine. The health care field will then truly enter the age of big data.

## Appendix

Multimedia Appendix 1 ICD-10-CM diagnosis code tutorial.

Multimedia Appendix 2 Detailed training and testing AUCs and F-measures for the 5-fold cross-validation test.

Multimedia Appendix 3 Detailed training and testing AUCs and F-measures for the real-world test.

## Abbreviations

AUC: area under the curve
CNN: convolutional neural network
GBM: gradient boosting machine
ICD-10-CM: International Classification of Diseases, Tenth Revision, Clinical Modification
NLP: natural language processing
SVM: support vector machine
RF: random forest
