# Supplementary material for: Artificial Intelligence Learning Semantics via External Resources for Classifying Diagnosis Codes in Discharge Notes
Source: J Med Internet Res. 2017 Nov 6;19(11):e380. doi: 10.2196/jmir.8344 (PMC5696581; doi:10.2196/jmir.8344)

A tutorial of ICD-10-CM diagnosis codes identification in free-text discharge note by word embedding and convolutional neural network

Chin Lin, Yu-Sheng Lou

National Defense Medical Center, Taipei, Taiwan, ROC

Background

Disease coding is a method for extracting structured information from free-text medical narratives. This structure disease information is useful in disease statistics and public health surveillance. The current methods for collecting this structured information usually involve manual identification, but manual identification of disease codes is laborious and costly. Moreover, time effectiveness is also important, so the automated disease code classification algorithm is important in future.

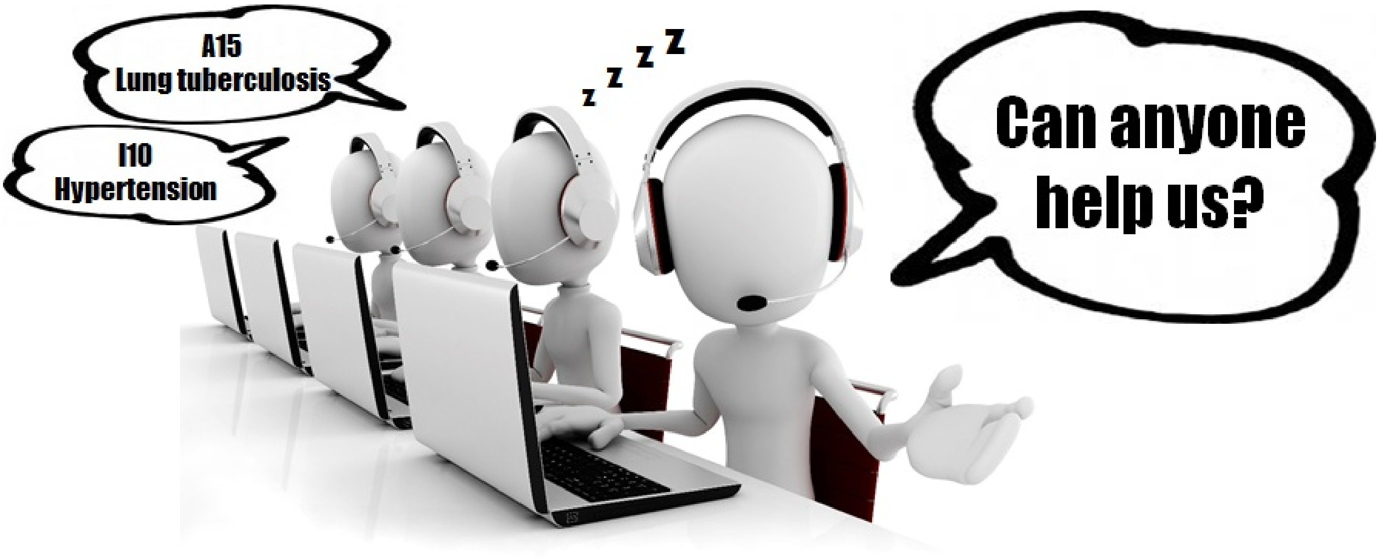

Traditionally, the automated disease code classification algorithm is based on two steps: (1) natural language processing (NLP) pipelines for feature engineering, and the popular method is to vectorize the documents based on “bag of words” model; (2) supervised machine learning models, such as support vector machine and random forests, are used to conduct classification task by these vectors. However, three problems limit traditional method: (1) incomplete medical dictionary, (2) emerging disease, and (3) curse of dimensionality.

We propose a NLP-free method for identifying ICD-10-CM diagnosis codes from free-text medical narratives. This method is based on word embedding and convolutional neural network (CNN). Word embedding is a feature-learning technique where vocabularies are mapped to vectors of real numbers, and the word vectors for similar concepts are likewise close in terms of cosine similarity and Euclidean distance. A free-text medical narratives can be converted into an N by M matrix through word embedding technology, where N is number of words among a document and M is the length of word embedding vector. CNN utilize layers with convolving filters that are applied to local features, and they can handle matrix input. Here, we will demonstrate this algorithm for ICD-10-CM diagnosis codes identification in free-text discharge note. The

wikipedia database is used to train a word embedding model, and discharge notes will be converted to a matrix for training CNN.

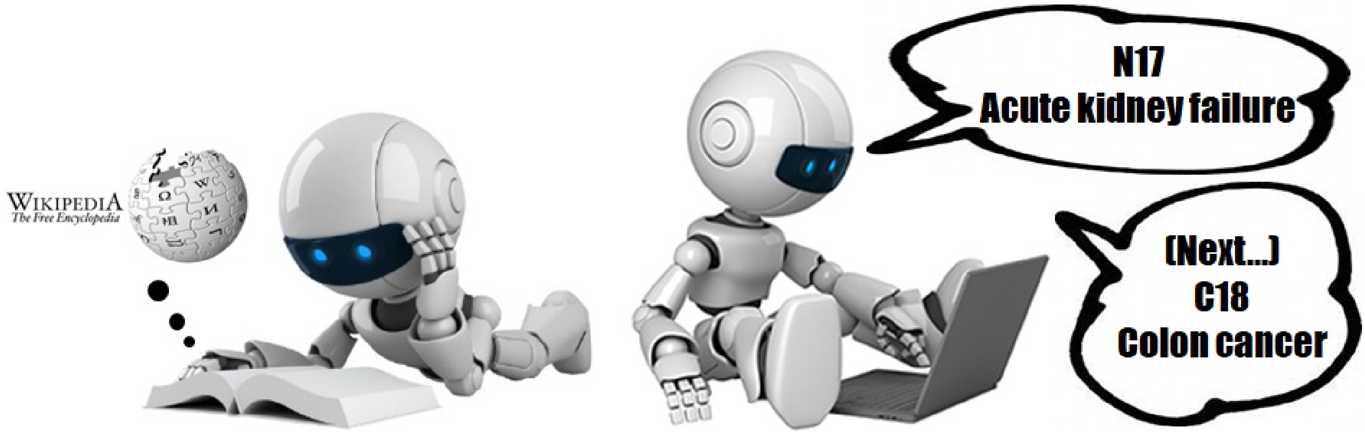

Diagram of processing nodes

In this tutorial, we propose a method combining word embedding and CNN for identifying the ICD-10 CM in discharge note. The diagram of processing nodes with with tags of codes is shown as following. We need to prepare a big text data (Wikipedia in our work), discharge notes, and their corresponding ICD-10 CM code in the begin. Then there are three steps for training an automated classification algorithm as following: (1) word embedding training, (2) converting free-text discharge note to a matrix, and (3) training a CNN. The implementation in R languge is shown as following section.

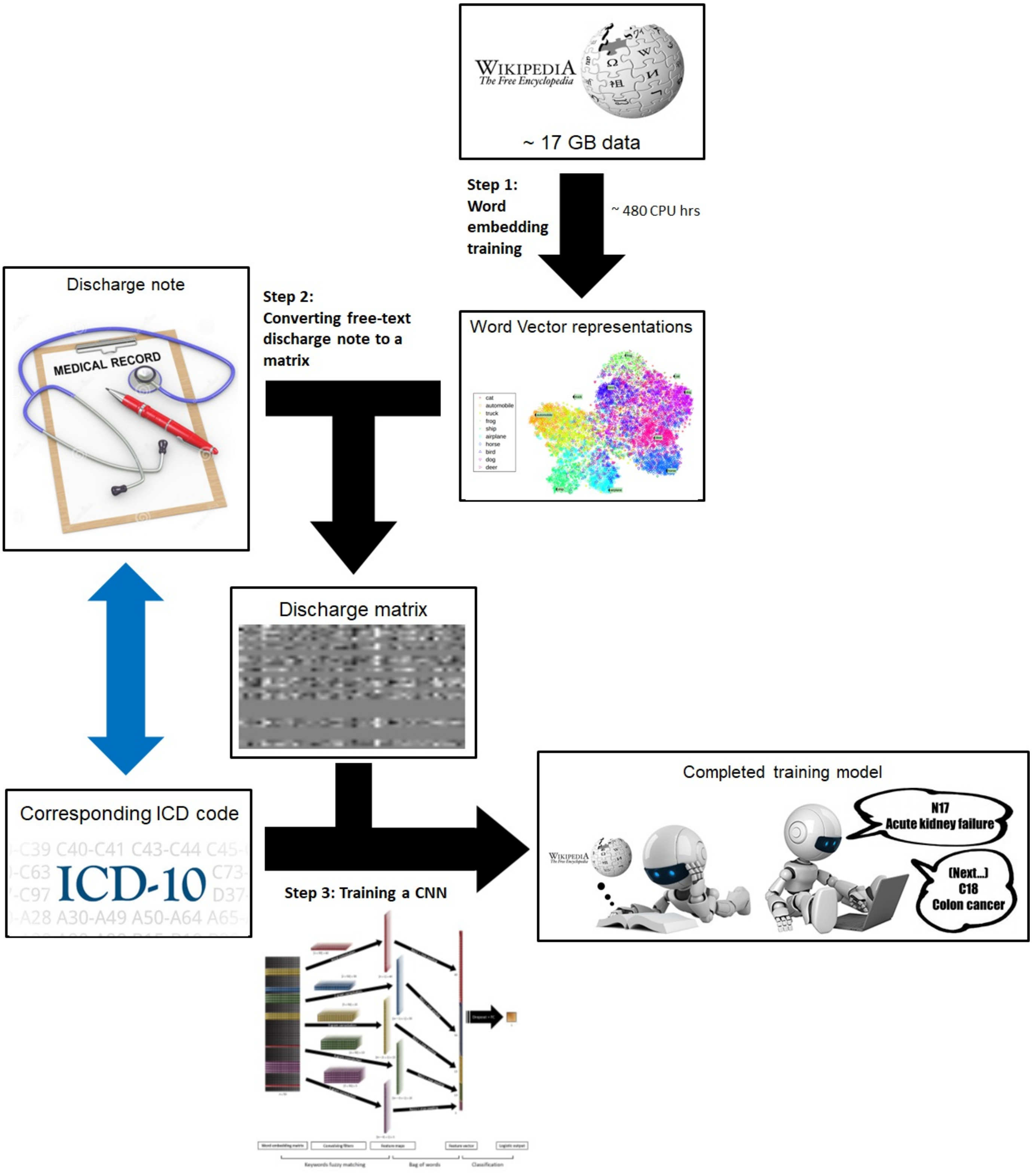

Implementation in R languge

1. Word embedding training

Word2Vec is currently the most popular word embedding algorithms. First, we need to a big text data for training . Wikipedia is an encyclopedia that is a written compendium of knowledge, and you can download them from **here**. Here, we demonstrate a simple word2vec training process by a smaller text data.

We need to download wordVectors package. This package can be downloaded from **github**. The main function for training word2vec models using an extended Jian Li's word2vec code; reads and writes the binary word2vec format so that you can import pre-trained models such as Google's; and provides tools for

reading only part of a model (rows or columns) so you can explore a model in memory-limited situations.

```
library(devtools)
install_github("bmschmidt/wordVectors")
```

Now we can use our smaller text data (download from **here**) for training our first word embedding model. This data is a cookbook and we can use following code to download it.

```
download.file("http://archive.lib.msu.edu/dinfo/feedingamerica/cookbook_text.zip","
cookbooks.zip")
unzip("cookbooks.zip",exdir="cookbooks")
```

We have many txt documents in “cookbooks” directory. Let us process them for further training.

```
library(wordVectors)
library(stringi)

prep_word2vec("cookbooks","cookbooks.txt",lowercase=T)
```

Training word2vec model (about 10-15 mins).

```
word.model = train_word2vec("cookbooks.txt", output="cookbook_vectors.bin", threads
= 3, vectors = 100, window=12)
```

Now we can ckeck the similarity of words.

```
nearest_to(word.model, word.model[["fish"]])
```

```
##         fish       salmon     pickerel      haddock        trout
## 3.330669e-16 2.056561e-01 2.097011e-01 2.280171e-01 2.289291e-01
##       smelts         pike    flounders         carp       turbot
## 2.384494e-01 2.422434e-01 2.458153e-01 2.472751e-01 2.546006e-01
```

Is it amazing? Of crouse, you can use your data for training word embedding model. The completed training model by wikipedia can be download from **here**, and we will use this embedding table in next step.

2. Converting free-text discharge note to a matrix

After word embedding model training finish, we need to build a pipeline for converting free-text discharge note to a matrix. Here, we only use an discharge note for example. You can apply this process to your data.

```r
#Example discharge note
example = "Adenocarcinoma of stomach with peritoneal carcinomatosis and massive asc
ite, stage IV under bidirection chemotherapy (neoadjuvant intraperitoneal-systemic
chemotherapy) with intraperitoneal paclitaxel 120mg (20151126, 20151201) and system
ic with Oxalip (20151127) and oral XELOX."

#Text process
text = tolower(example)
text = gsub("\n", "@@@@@", text, fixed = TRUE)
text = gsub("\r", "@@@@@", text, fixed = TRUE)
text = gsub("[ :,;-]", "@", text)
text = gsub("(", "@", text, fixed = TRUE)
text = gsub(")", "@", text, fixed = TRUE)
text = gsub("/", "@", text, fixed = TRUE)
text = strsplit(text, split = ".", fixed = TRUE)[[1]]
text = paste(text, collapse = "@@@@@")
text = strsplit(text, split = "@", fixed = TRUE)[[1]]

#Show result
text
```

```
##  [1] "adenocarcinoma"  "of"              "stomach"
##  [4] "with"            "peritoneal"      "carcinomatosis"
##  [7] "and"             "massive"         "ascite"
## [10] ""                "stage"           "iv"
## [13] "under"           "bidirection"     "chemotherapy"
## [16] ""                "neoadjuvant"     "intraperitoneal"
## [19] "systemic"        "chemotherapy"    ""
## [22] "with"            "intraperitoneal" "paclitaxel"
## [25] "120mg"           ""                "20151126"
## [28] ""                "20151201"        ""
## [31] "and"             "systemic"        "with"
## [34] "oxalip"          ""                "20151127"
## [37] ""                "and"             "oral"
## [40] "xelox"
```

We can find the example discharge note has been converted to a word vector. Now we need to use the completed training word embedding model (download from **here**) for converting word vector to a matrix.

```r
#Read word embedding model
library(data.table)
library(magrittr)
library(dplyr)
library(plyr)

word.data = fread("wikipedia word2vec.txt", header = FALSE, showProgress = FALSE)
words.ref = word.data %>% select(V1) %>% setDF %>% .[,1] %>% as.character
words.matrix = word.data %>% select(-V1) %>% setDF %>% as.matrix

#Alignment
TEXT.ARRAY = matrix(0, nrow = length(text), ncol = 50)
for (i in 1:length(text)) {
  if (text[i]!="") {
    pos = which(words.ref == text[i])
    if (length(pos)==1) {
      TEXT.ARRAY[i,] = words.matrix[pos,]
    }
  }
}
```

Let us visualize this matrix!

```r
library(imager)
img = TEXT.ARRAY
img[img>2] = 2
img[img<-2] = -2
plot(as.cimg(t(img)))
```

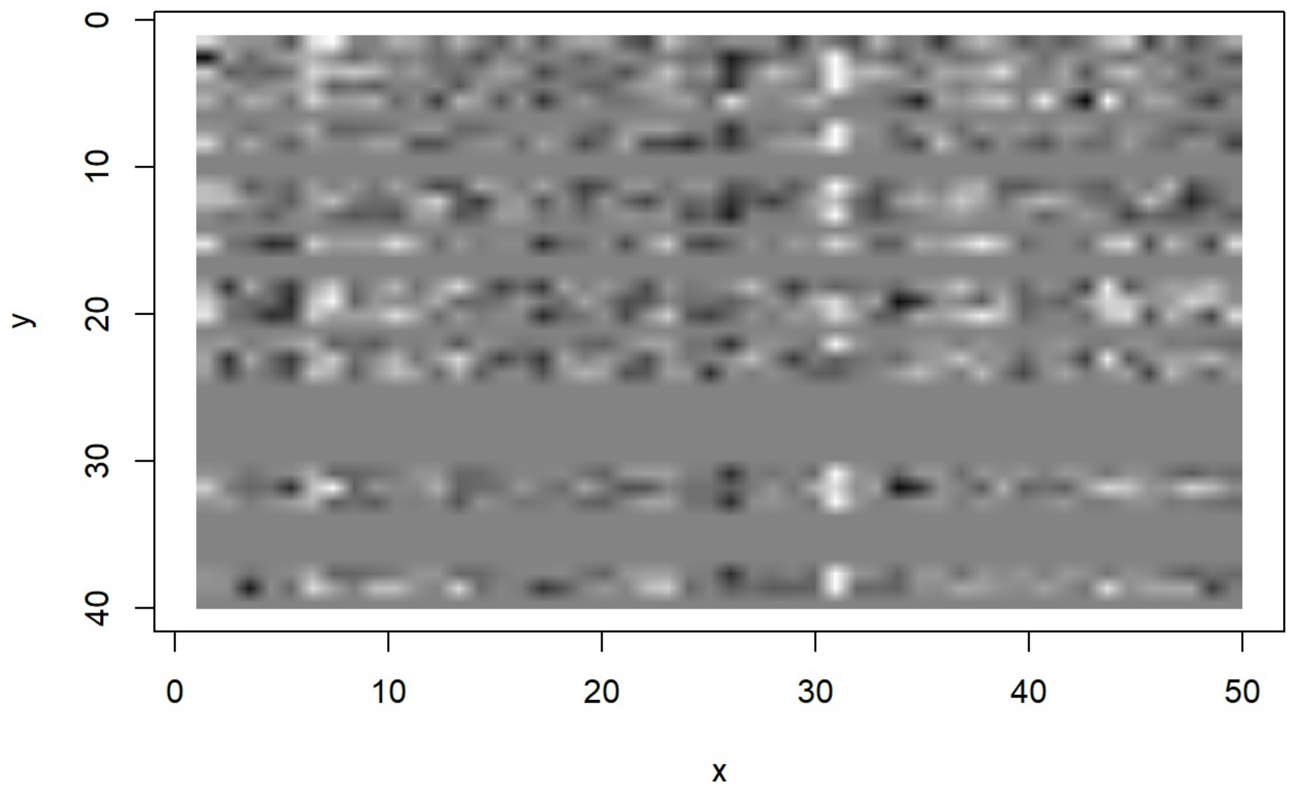

3. Training a CNN

Let us download 5,000 labeled discharge note after converting process from **here**. This data include 2 objects: **ARRAY** and **LABEL**. Where **ARRAY** is a 4-D array representing 5,000 discharge notes, and **LABEL** recorded which discharge note mentions neoplasms(C00-D49). There are 1,640 neoplasms discharge notes. Here we can divide it into a training set and testing set.

- All matrices have been converted to 100 rows, and additional part have been filled in 0. This meaning the all of our example discharge notes are less than 100 words.

```
load("ICD10.RData")

Train.X.array = ARRAY[,,1:3000]
dim(Train.X.array) = c(100, 50, 1, 3000)
Train.Y = LABEL[1:3000]

Test.X.array = ARRAY[,,3001:5000]
dim(Test.X.array) = c(100, 50, 1, 2000)
Test.Y = LABEL[3001:5000]
```

We need to download MxNet package. MXNet for R is available for both CPUs and GPUs. For Windows users, MXNet provides prebuilt binary packages. You can install the package directly in the R console (CPU version).

```
cran <- getOption("repos")
cran["dmlc"] <- "https://s3-us-west-2.amazonaws.com/apache-mxnet/R/CRAN/"
options(repos = cran)
install.packages("mxnet")
```

Now let us refer this to design our CNN.

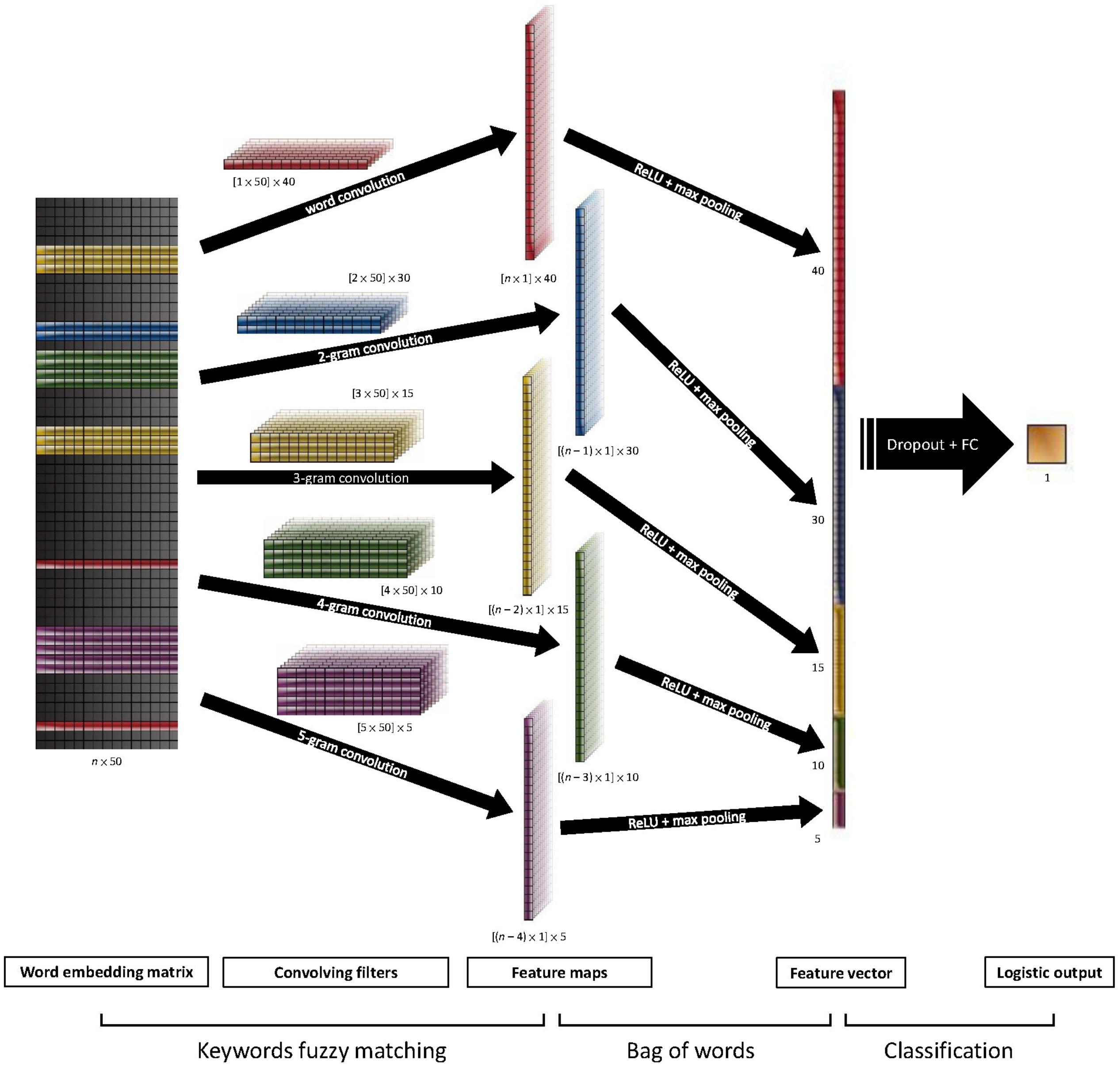
[1 x 50] x 40
word convolution
[n x 1] x 40
ReLU + max pooling
40
[2 x 50] x 30
2-gram convolution
[(n – 1) x 1] x 30
ReLU + max pooling
30
[3 x 50] x 15
3-gram convolution
[(n – 2) x 1] x 15
ReLU + max pooling
15
[4 x 50] x 10
4-gram convolution
[(n – 3) x 1] x 10
ReLU + max pooling
10
[5 x 50] x 5
5-gram convolution
[(n – 4) x 1] x 5
ReLU + max pooling
5
n x 50
Dropout + FC
1
Word embedding matrix
Convolving filters
Feature maps
Feature vector
Logistic output
Keywords fuzzy matching
Bag of words
Classification

```r
library(mxnet)

get_symbol_textcnn <- function(num_outcome = 1, filter_sizes = 1:5, num_filter = c(40, 30, 15, 10, 5), Seq.length = 100, word.dimation = 50, dropout = 0.5) {

  data <- mx.symbol.Variable('data')

  concat_lst <- NULL

  for (i in 1:length(filter_sizes)) {
    convi <- mx.symbol.Convolution(data = data, kernel = c(filter_sizes[i], word.dimation), pad = c(filter_sizes[i]-1, 0), num_filter = num_filter[i], name = paste0('conv', i))
    relui <- mx.symbol.Activation(data = convi, act_type = "relu", name = paste0('relu', i))
    pooli <- mx.symbol.Pooling(data = relui, pool_type = "max", kernel = c(Seq.length + filter_sizes[i] - 1, 1), stride = c(1, 1), name = paste0('pool', i))
    concat_lst = append(concat_lst, pooli)
  }

  concat_lst$num.args = length(filter_sizes)

  h_pool = mxnet:::mx.varg.symbol.Concat(concat_lst)

  # dropout layer

  if (dropout > 0) {
    h_drop = mx.symbol.Dropout(data = h_pool, p = dropout)
  } else {
    h_drop = h_pool
  }

  # fully connected layer

  cls_weight = mx.symbol.Variable('cls_weight')
  cls_bias = mx.symbol.Variable('cls_bias')

  fc = mx.symbol.FullyConnected(data = h_drop, weight = cls_weight, bias = cls_bias, num_hidden = num_outcome)
  lr = mx.symbol.LogisticRegressionOutput(fc, name='lr')

  return(lr)
}
```

The cross-entropy is used for loss function. We need to define this function as following.

```r
my.eval.metric.CE <- mx.metric.custom(
  name = "Cross-Entropy (CE)",
  function(real, pred) {
    real1 = as.numeric(real)
    pred1 = as.numeric(pred)
    pred1[pred1 <= 1e-6] = 1e-6
    pred1[pred1 >= 1 - 1e-6] = 1 - 1e-6
    return(-mean(real1 * log(pred1) + (1 - real1) * log(1 - pred1), na.rm = TRUE))
  }
)
```

Now we are finally able to train our first model.

```r
n.cpu <- 4
device.cpu <- lapply(0:(n.cpu-1), function(i) {mx.cpu(i)})

mx.set.seed(0)

cnn.model = mx.model.FeedForward.create(get_symbol_textcnn(),
                                        X = Train.X.array, y = Train.Y,
                                        ctx = device.cpu, num.round = 20,
                                        array.batch.size = 100, learning.rate = 0.
05,
                                        momentum = 0.9, wd = 0.00001,
                                        eval.metric = my.eval.metric.CE)
```

```
## Auto-select kvstore type = local_update_cpu
## Start training with 4 devices
## [1] Train-Cross-Entropy (CE)=0.646637871900186
## [2] Train-Cross-Entropy (CE)=0.557748752890724
## [3] Train-Cross-Entropy (CE)=0.371177842529053
## [4] Train-Cross-Entropy (CE)=0.302762303566566
## [5] Train-Cross-Entropy (CE)=0.263237404758913
## [6] Train-Cross-Entropy (CE)=0.250432130185339
## [7] Train-Cross-Entropy (CE)=0.234207048904955
## [8] Train-Cross-Entropy (CE)=0.237682273764665
## [9] Train-Cross-Entropy (CE)=0.201103999045526
## [10] Train-Cross-Entropy (CE)=0.205322830078845
## [11] Train-Cross-Entropy (CE)=0.194253797400228
## [12] Train-Cross-Entropy (CE)=0.170224793233577
## [13] Train-Cross-Entropy (CE)=0.163891660067964
## [14] Train-Cross-Entropy (CE)=0.181323002454327
## [15] Train-Cross-Entropy (CE)=0.18229354797829
## [16] Train-Cross-Entropy (CE)=0.158645556911646
## [17] Train-Cross-Entropy (CE)=0.156562428821528
## [18] Train-Cross-Entropy (CE)=0.141004996980063
## [19] Train-Cross-Entropy (CE)=0.134122406101353
## [20] Train-Cross-Entropy (CE)=0.13564977252312
```

Let us use this model to predict testing set, and use receiver operating characteristic (ROC) curve to assess our model performance.

```r
library(pROC)
pred.y = predict(cnn.model, Test.X.array)
ROC.test = roc(response = Test.Y, predictor = as.numeric(pred.y))
print(auc(ROC.test))
```

```
## Area under the curve: 0.9808
```

It is amazing that area under the curve is more than 0.98 using only 3,000 sample to train our model. We can use following code to save our model.

```r
mx.model.save(cnn.model, "Neoplasms CNN", iteration = 1)
```

If you want to use our pre-training Neoplasms CNN model, you can download from **here**. And now we can use this model to predict testing data again.

```r
pre_training.cnn = mx.model.load("Neoplasms CNN", iteration = 0)
pred.y = predict(pre_training.cnn, Test.X.array)
ROC.test = roc(response = Test.Y, predictor = as.numeric(pred.y))
print(auc(ROC.test))
```

```
## Area under the curve: 0.9952
```

The area under the curve of completed model in testing set is more than 0.995! Now it’s your turn!

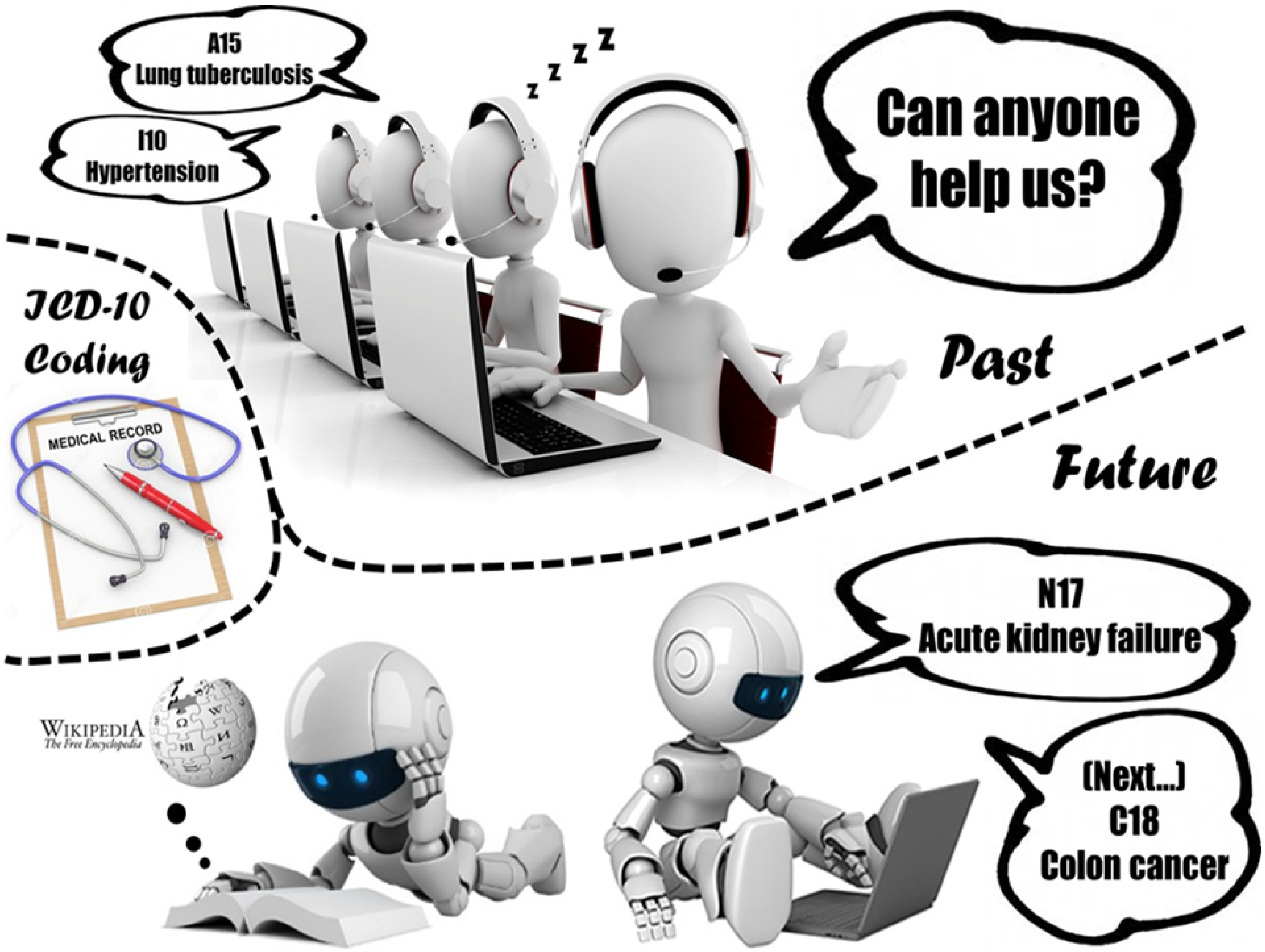

Supplement: Multimedia Appendix 1 [file jmir_v19i11e380_app1.pdf]
