# Supplementary material for: Artificial Intelligence Learning Semantics via External Resources for Classifying Diagnosis Codes in Discharge Notes
Source: J Med Internet Res. 2017 Nov 6;19(11):e380. doi: 10.2196/jmir.8344 (PMC5696581; doi:10.2196/jmir.8344)
Supplement: Multimedia Appendix 2 [file jmir_v19i11e380_app2.pdf]

Table S1-1. Training and testing AUC of A00-B99 (Certain infectious and parasitic diseases) in 5-fold cross validation test.

	Training set		Testing set	
	**AUC**	**F-measure**	**AUC**	**F-measure**
**Traditional pipeline**				
NLP + SVM (linear)	0.9974	0.9843	0.9736	0.9239
NLP + SVM (polynomial)	0.6813	0.2854	0.6873	0.3019
NLP + SVM (radial basis)	0.9606	0.7787	0.9544	0.7795
NLP + SVM (sigmoid)	0.9606	0.7786	0.9544	0.7791
NLP + RF	1.0000	0.9956	0.9809	0.9401
NLP + GBM	0.9998	0.9950	0.9758	0.9338
**Proposed pipeline**				
GloVe + CNN	0.9970	0.9831	0.9885	0.9582

Table S1-2. Training and testing AUC of C00-D49 (Neoplasms) in 5-fold cross validation test.

	Training set		Testing set	
	**AUC**	**F-measure**	**AUC**	**F-measure**
**Traditional pipeline**				
NLP + SVM (linear)	0.9972	0.9738	0.9842	0.9396
NLP + SVM (polynomial)	0.7704	0.6125	0.7664	0.6094
NLP + SVM (radial basis)	0.9432	0.8396	0.9398	0.8354
NLP + SVM (sigmoid)	0.9285	0.8313	0.9245	0.8274
NLP + RF	0.9996	0.9833	0.9848	0.9446
NLP + GBM	0.9995	0.9812	0.9843	0.9420
**Proposed pipeline**				
GloVe + CNN	0.9952	0.9662	0.9910	0.9523

Table S1-3. Training and testing AUC of D50-D89 (Diseases of the blood and blood-forming organs and certain disorders involving the immune mechanism) in 5-fold cross validation test.

	Training set		Testing set	
	**AUC**	**F-measure**	**AUC**	**F-measure**
**Traditional pipeline**				
NLP + SVM (linear)	0.9985	0.9873	0.9811	0.9329
NLP + SVM (polynomial)	0.9139	0.5149	0.8885	0.4688
NLP + SVM (radial basis)	0.9873	0.8748	0.9757	0.8640
NLP + SVM (sigmoid)	0.9871	0.8746	0.9753	0.8643
NLP + RF	0.9999	0.9931	0.9782	0.9515
NLP + GBM	0.9997	0.9924	0.9780	0.9490
**Proposed pipeline**				
GloVe + CNN	0.9984	0.9930	0.9829	0.9578

Table S1-4. Training and testing AUC of E00-E89 (Endocrine, nutritional and metabolic diseases) in 5-fold cross validation test.

	Training set		Testing set	
	**AUC**	**F-measure**	**AUC**	**F-measure**
**Traditional pipeline**				
NLP + SVM (linear)	0.9982	0.9905	0.9848	0.9612
NLP + SVM (polynomial)	0.7032	0.4370	0.7017	0.4415
NLP + SVM (radial basis)	0.9440	0.7513	0.9382	0.7510
NLP + SVM (sigmoid)	0.9438	0.7512	0.9382	0.7510
NLP + RF	1.0000	0.9961	0.9856	0.9690
NLP + GBM	0.9999	0.9959	0.9856	0.9691
**Proposed pipeline**				
GloVe + CNN	0.9976	0.9829	0.9889	0.9670

Table S1-5. Training and testing AUC of F01-F99 (Mental, Behavioral and Neurodevelopmental disorders) in 5-fold cross validation test.

	Training set		Testing set	
	**AUC**	**F-measure**	**AUC**	**F-measure**
**Traditional pipeline**				
NLP + SVM (linear)	0.9999	0.9966	0.9928	0.9711
NLP + SVM (polynomial)	0.9841	0.8959	0.9522	0.8215
NLP + SVM (radial basis)	0.9630	0.8510	0.9644	0.8427
NLP + SVM (sigmoid)	0.9398	0.7774	0.9395	0.7625
NLP + RF	1.0000	0.9982	0.9898	0.9766
NLP + GBM	1.0000	0.9985	0.9909	0.9762
**Proposed pipeline**				
GloVe + CNN	0.9998	0.9972	0.9964	0.9865

Table S1-6. Training and testing AUC of G00-G99 (Diseases of the nervous system) in 5-fold cross validation test.

	Training set		Testing set	
	**AUC**	**F-measure**	**AUC**	**F-measure**
**Traditional pipeline**				
NLP + SVM (linear)	0.9981	0.9787	0.9614	0.8722
NLP + SVM (polynomial)	0.9207	0.5008	0.8421	0.3518
NLP + SVM (radial basis)	0.9754	0.8041	0.9644	0.7478
NLP + SVM (sigmoid)	0.9748	0.8032	0.9632	0.7455
NLP + RF	1.0000	0.9933	0.9739	0.9009
NLP + GBM	0.9997	0.9898	0.9727	0.8867

**Proposed pipeline**				
GloVe + CNN	0.9991	0.9942	0.9800	0.9237

Table S1-7. Training and testing AUC of H00-H59 (Diseases of the eye and adnexa) in 5-fold cross validation test.

	**Training set**		**Testing set**	
	**AUC**	**F-measure**	**AUC**	**F-measure**
**Traditional pipeline**				
NLP + SVM (linear)	0.9999	0.9946	0.9730	0.9202
NLP + SVM (polynomial)	0.9886	0.8328	0.9358	0.7700
NLP + SVM (radial basis)	0.9554	0.8880	0.9451	0.8796
NLP + SVM (sigmoid)	0.9552	0.8876	0.9450	0.8796
NLP + RF	1.0000	0.9904	0.9789	0.9463
NLP + GBM	1.0000	0.9969	0.9762	0.9371
**Proposed pipeline**				
GloVe + CNN	0.9998	0.9981	0.9883	0.9466

Table S1-8. Training and testing AUC of H60-H95 (Diseases of the ear and mastoid process) in 5-fold cross validation test.

	**Training set**		**Testing set**	
	**AUC**	**F-measure**	**AUC**	**F-measure**
**Traditional pipeline**				
NLP + SVM (linear)	0.9999	0.9948	0.9862	0.9299
NLP + SVM (polynomial)	0.9750	0.6967	0.9071	0.5696
NLP + SVM (radial basis)	0.9908	0.9074	0.9787	0.8820
NLP + SVM (sigmoid)	0.9900	0.9079	0.9781	0.8823
NLP + RF	1.0000	0.9870	0.9741	0.9538
NLP + GBM	1.0000	0.9948	0.9822	0.9270
**Proposed pipeline**				
GloVe + CNN	0.9999	0.9995	0.9827	0.9480

Table S1-9. Training and testing AUC of I00-I99 (Diseases of the circulatory system) in 5-fold cross validation test.

	**Training set**		**Testing set**	
	**AUC**	**F-measure**	**AUC**	**F-measure**
**Traditional pipeline**				
NLP + SVM (linear)	0.9968	0.9863	0.9835	0.9566
NLP + SVM (polynomial)	0.9184	0.7502	0.9098	0.7397
NLP + SVM (radial basis)	0.9509	0.8558	0.9530	0.8599
NLP + SVM (sigmoid)	0.9416	0.8190	0.9443	0.8238
NLP + RF	0.9999	0.9951	0.9857	0.9644

NLP + GBM	0.9998	0.9935	0.9860	0.9650
**Proposed pipeline**				
GloVe + CNN	0.9949	0.9730	0.9881	0.9588

Table S1-10. Training and testing AUC of J00-J99 (Diseases of the respiratory system) in 5-fold cross validation test.

	Training set		Testing set	
	**AUC**	**F-measure**	**AUC**	**F-measure**
**Traditional pipeline**				
NLP + SVM (linear)	0.9989	0.9913	0.9796	0.9430
NLP + SVM (polynomial)	0.6150	0.2507	0.5980	0.2448
NLP + SVM (radial basis)	0.9698	0.8127	0.9651	0.8067
NLP + SVM (sigmoid)	0.9700	0.8125	0.9652	0.8062
NLP + RF	1.0000	0.9980	0.9848	0.9534
NLP + GBM	0.9998	0.9962	0.9857	0.9527
**Proposed pipeline**				
GloVe + CNN	0.9990	0.9928	0.9893	0.9555

Table S1-11. Training and testing AUC of K00-K95 (Diseases of the digestive system) in 5-fold cross validation test.

	Training set		Testing set	
	**AUC**	**F-measure**	**AUC**	**F-measure**
**Traditional pipeline**				
NLP + SVM (linear)	0.9960	0.9801	0.9711	0.9245
NLP + SVM (polynomial)	0.9176	0.6598	0.8839	0.6259
NLP + SVM (radial basis)	0.9532	0.8104	0.9468	0.8125
NLP + SVM (sigmoid)	0.9531	0.8101	0.9468	0.8122
NLP + RF	0.9999	0.9969	0.9838	0.9445
NLP + GBM	0.9997	0.9959	0.9808	0.9413
**Proposed pipeline**				
GloVe + CNN	0.9971	0.9783	0.9853	0.9405

Table S1-12. Training and testing AUC of L00-L99 (Diseases of the skin and subcutaneous tissue) in 5-fold cross validation test.

	Training set		Testing set	
	**AUC**	**F-measure**	**AUC**	**F-measure**
**Traditional pipeline**				
NLP + SVM (linear)	0.9975	0.9759	0.9587	0.8490
NLP + SVM (polynomial)	0.9435	0.5695	0.8677	0.4403
NLP + SVM (radial basis)	0.9714	0.7656	0.9510	0.7157
NLP + SVM (sigmoid)	0.9704	0.7646	0.9502	0.7130

NLP + RF	1.0000	0.9903	0.9538	0.8679
NLP + GBM	0.9993	0.9849	0.9441	0.8525
**Proposed pipeline**				
GloVe + CNN	0.9990	0.9944	0.9729	0.9158

Table S1-13. Training and testing AUC of M00-M99 (Diseases of the musculoskeletal system and connective tissue) in 5-fold cross validation test.

	**Training set**		**Testing set**	
	**AUC**	**F-measure**	**AUC**	**F-measure**
**Traditional pipeline**				
NLP + SVM (linear)	0.9953	0.9731	0.9691	0.9104
NLP + SVM (polynomial)	0.9430	0.6843	0.9049	0.6222
NLP + SVM (radial basis)	0.9558	0.8018	0.9493	0.7855
NLP + SVM (sigmoid)	0.9559	0.8018	0.9493	0.7864
NLP + RF	0.9999	0.9938	0.9773	0.9227
NLP + GBM	0.9999	0.9921	0.9734	0.9175
**Proposed pipeline**				
GloVe + CNN	0.9957	0.9766	0.9794	0.9282

Table S1-14. Training and testing AUC of N00-N99 (Diseases of the genitourinary system) in 5-fold cross validation test.

	**Training set**		**Testing set**	
	**AUC**	**F-measure**	**AUC**	**F-measure**
**Traditional pipeline**				
NLP + SVM (linear)	0.9966	0.9810	0.9713	0.9169
NLP + SVM (polynomial)	0.6389	0.3221	0.6271	0.3166
NLP + SVM (radial basis)	0.9617	0.8060	0.9552	0.7907
NLP + SVM (sigmoid)	0.9617	0.8060	0.9553	0.7908
NLP + RF	1.0000	0.9971	0.9813	0.9436
NLP + GBM	0.9998	0.9953	0.9790	0.9362
**Proposed pipeline**				
GloVe + CNN	0.9972	0.9809	0.9843	0.9449

Table S1-15. Training and testing AUC of O00-O9A (Pregnancy, childbirth and the puerperium) in 5-fold cross validation test.

	**Training set**		**Testing set**	
	**AUC**	**F-measure**	**AUC**	**F-measure**
**Traditional pipeline**				
NLP + SVM (linear)	0.9999	0.9987	0.9973	0.9907
NLP + SVM (polynomial)	0.9947	0.8434	0.9916	0.8444
NLP + SVM (radial basis)	0.9981	0.9751	0.9975	0.9718

NLP + SVM (sigmoid)	0.9981	0.9751	0.9975	0.9718
NLP + RF	1.0000	0.9991	0.9977	0.9931
NLP + GBM	1.0000	0.9987	0.9981	0.9923
**Proposed pipeline**				
GloVe + CNN	1.0000	0.9995	0.9989	0.9937

Table S1-16. Training and testing AUC of P00-P96 (Certain conditions originating in the perinatal period) in 5-fold cross validation test.

	**Training set**		**Testing set**	
	**AUC**	**F-measure**	**AUC**	**F-measure**
**Traditional pipeline**				
NLP + SVM (linear)	0.9995	0.9805	0.9852	0.9371
NLP + SVM (polynomial)	0.9944	0.8192	0.9875	0.7613
NLP + SVM (radial basis)	0.9938	0.8900	0.9928	0.8939
NLP + SVM (sigmoid)	0.9938	0.8911	0.9928	0.8939
NLP + RF	0.9997	0.9767	0.9869	0.9417
NLP + GBM	0.9995	0.9753	0.9886	0.9434
**Proposed pipeline**				
GloVe + CNN	0.9998	0.9988	0.9965	0.9777

Table S1-17. Training and testing AUC of Q00-Q99 (Congenital malformations, deformations and chromosomal abnormalities) in 5-fold cross validation test.

	**Training set**		**Testing set**	
	**AUC**	**F-measure**	**AUC**	**F-measure**
**Traditional pipeline**				
NLP + SVM (linear)	0.9966	0.9385	0.9072	0.7078
NLP + SVM (polynomial)	0.8937	0.2868	0.7462	0.1166
NLP + SVM (radial basis)	0.9752	0.8000	0.9061	0.5919
NLP + SVM (sigmoid)	0.9717	0.7649	0.9074	0.5827
NLP + RF	1.0000	0.9564	0.8731	0.7220
NLP + GBM	0.9988	0.9476	0.8706	0.6503
**Proposed pipeline**				
GloVe + CNN	0.9997	0.9981	0.9025	0.7590

Table S1-18. Training and testing AUC of R00-R99 (Symptoms, signs and abnormal clinical and laboratory findings, not elsewhere classified) in 5-fold cross validation test.

	**Training set**		**Testing set**	
	**AUC**	**F-measure**	**AUC**	**F-measure**
**Traditional pipeline**				
NLP + SVM (linear)	0.9901	0.9160	0.9034	0.7278
NLP + SVM (polynomial)	0.9021	0.5645	0.7922	0.4037

NLP + SVM (radial basis)	0.9515	0.7130	0.9073	0.6221
NLP + SVM (sigmoid)	0.9496	0.7091	0.9045	0.6199
NLP + RF	0.9999	0.9867	0.9192	0.7454
NLP + GBM	0.9994	0.9873	0.9221	0.7524
**Proposed pipeline**				
GloVe + CNN	0.9943	0.9745	0.9434	0.8538

Table S1-19. Training and testing AUC of S00-T88 (Injury, poisoning and certain other consequences of external causes) in 5-fold cross validation test.

	**Training set**		**Testing set**	
	**AUC**	**F-measure**	**AUC**	**F-measure**
**Traditional pipeline**				
NLP + SVM (linear)	0.9907	0.9474	0.9395	0.8471
NLP + SVM (polynomial)	0.9317	0.6363	0.8942	0.5787
NLP + SVM (radial basis)	0.9139	0.6928	0.9069	0.6914
NLP + SVM (sigmoid)	0.9136	0.6926	0.9065	0.6913
NLP + RF	0.9998	0.9895	0.9512	0.8524
NLP + GBM	0.9997	0.9861	0.9445	0.8499
**Proposed pipeline**				
GloVe + CNN	0.9963	0.9809	0.9649	0.9082

Table S1-20. Training and testing AUC of V00-Y99 (External causes of morbidity) in 5-fold cross validation test.

	**Training set**		**Testing set**	
	**AUC**	**F-measure**	**AUC**	**F-measure**
**Traditional pipeline**				
NLP + SVM (linear)	0.9767	0.5861	0.7917	0.1042
NLP + SVM (polynomial)	0.7296	0.1040	0.5519	0.0316
NLP + SVM (radial basis)	0.9071	0.4665	0.7066	0.0916
NLP + SVM (sigmoid)	0.8643	0.3493	0.6811	0.0840
NLP + RF	0.9998	0.9199	0.7262	0.0946
NLP + GBM	0.9997	0.9539	0.7053	0.1454
**Proposed pipeline**				
GloVe + CNN	0.9996	0.9927	0.8220	0.4434

Table S1-21. Training and testing AUC of Z00-Z99 (Factors influencing health status and contact with health services) in 5-fold cross validation test.

	**Training set**		**Testing set**	
	**AUC**	**F-measure**	**AUC**	**F-measure**
**Traditional pipeline**				
NLP + SVM (linear)	0.9653	0.8921	0.9036	0.8065

NLP + SVM (polynomial)	0.7569	0.5569	0.7473	0.5443
NLP + SVM (radial basis)	0.8648	0.6827	0.8639	0.6789
NLP + SVM (sigmoid)	0.8645	0.6819	0.8637	0.6787
NLP + RF	0.9988	0.9777	0.9305	0.8230
NLP + GBM	0.9980	0.9722	0.9187	0.8313
**Proposed pipeline**				
GloVe + CNN	0.9647	0.9036	0.9348	0.8609
