# Supplementary material for: Artificial Intelligence Learning Semantics via External Resources for Classifying Diagnosis Codes in Discharge Notes
Source: J Med Internet Res. 2017 Nov 6;19(11):e380. doi: 10.2196/jmir.8344 (PMC5696581; doi:10.2196/jmir.8344)
Supplement: Multimedia Appendix 3 [file jmir_v19i11e380_app3.pdf]

**Table S2-1. Training and testing AUC of A00-B99 (Certain infectious and parasitic diseases) in real-world situation.**

| | Training set | | Testing set | |
|---|---|---|---|---|
| | **AUC** | **F-measure** | **AUC** | **F-measure** |
| **Traditional pipeline** | | | | |
| NLP + SVM (linear) | 0.9952 | 0.9768 | 0.9716 | 0.9289 |
| NLP + SVM (polynomial) | 0.9350 | 0.6582 | 0.9263 | 0.6578 |
| NLP + SVM (radial basis) | 0.9581 | 0.7783 | 0.9603 | 0.7861 |
| NLP + SVM (sigmoid) | 0.9581 | 0.7783 | 0.9603 | 0.7859 |
| NLP + RF | 1.0000 | 0.9938 | 0.9784 | 0.9440 |
| NLP + GBM | 0.9998 | 0.9921 | 0.9789 | 0.9435 |
| **Proposed pipeline** | | | | |
| GloVe + CNN | 0.9974 | 0.9840 | 0.9858 | 0.9568 |

**Table S2-2. Training and testing AUC of C00-D49 (Neoplasms) in real-world situation.**

| | Training set | | Testing set | |
|---|---|---|---|---|
| | **AUC** | **F-measure** | **AUC** | **F-measure** |
| **Traditional pipeline** | | | | |
| NLP + SVM (linear) | 0.9959 | 0.9679 | 0.9834 | 0.9240 |
| NLP + SVM (polynomial) | 0.7684 | 0.6116 | 0.7510 | 0.6270 |
| NLP + SVM (radial basis) | 0.9533 | 0.8492 | 0.9436 | 0.8388 |
| NLP + SVM (sigmoid) | 0.9419 | 0.8370 | 0.9309 | 0.8334 |
| NLP + RF | 0.9995 | 0.9830 | 0.9846 | 0.9285 |
| NLP + GBM | 0.9990 | 0.9775 | 0.9852 | 0.9246 |
| **Proposed pipeline** | | | | |
| GloVe + CNN | 0.9956 | 0.9666 | 0.9915 | 0.9541 |

**Table S2-3. Training and testing AUC of D50-D89 (Diseases of the blood and blood-forming organs and certain disorders involving the immune mechanism) in real-world situation.**

| | Training set | | Testing set | |
|---|---|---|---|---|
| | **AUC** | **F-measure** | **AUC** | **F-measure** |
| **Traditional pipeline** | | | | |
| NLP + SVM (linear) | 0.9974 | 0.9828 | 0.9695 | 0.9266 |
| NLP + SVM (polynomial) | 0.9518 | 0.6698 | 0.9273 | 0.6144 |
| NLP + SVM (radial basis) | 0.9827 | 0.8697 | 0.9708 | 0.8525 |
| NLP + SVM (sigmoid) | 0.9825 | 0.8694 | 0.9706 | 0.8523 |
| NLP + RF | 0.9999 | 0.9919 | 0.9782 | 0.9535 |
| NLP + GBM | 0.9996 | 0.9887 | 0.9750 | 0.9512 |
| **Proposed pipeline** | | | | |
| GloVe + CNN | 0.9982 | 0.9910 | 0.9829 | 0.9631 |

**Table S2-4. Training and testing AUC of E00-E89 (Endocrine, nutritional and metabolic diseases) in real-world situation.**

| | Training set | | Testing set | |
|---|---|---|---|---|
| | AUC | F-measure | AUC | F-measure |
| **Traditional pipeline** | | | | |
| NLP + SVM (linear) | 0.9970 | 0.9866 | 0.9876 | 0.9657 |
| NLP + SVM (polynomial) | 0.9096 | 0.7079 | 0.8970 | 0.6930 |
| NLP + SVM (radial basis) | 0.9498 | 0.7878 | 0.9501 | 0.7876 |
| NLP + SVM (sigmoid) | 0.9414 | 0.7511 | 0.9414 | 0.7493 |
| NLP + RF | 1.0000 | 0.9969 | 0.9893 | 0.9756 |
| NLP + GBM | 0.9998 | 0.9938 | 0.9891 | 0.9748 |
| **Proposed pipeline** | | | | |
| GloVe + CNN | 0.9970 | 0.9803 | 0.9906 | 0.9710 |

**Table S2-5. Training and testing AUC of F01-F99 (Mental, Behavioral and Neurodevelopmental disorders) in real-world situation.**

| | Training set | | Testing set | |
|---|---|---|---|---|
| | AUC | F-measure | AUC | F-measure |
| **Traditional pipeline** | | | | |
| NLP + SVM (linear) | 0.9998 | 0.9955 | 0.9875 | 0.9671 |
| NLP + SVM (polynomial) | 0.9747 | 0.8755 | 0.9481 | 0.8206 |
| NLP + SVM (radial basis) | 0.9811 | 0.8952 | 0.9791 | 0.8909 |
| NLP + SVM (sigmoid) | 0.9616 | 0.8465 | 0.9625 | 0.8355 |
| NLP + RF | 1.0000 | 0.9972 | 0.9860 | 0.9697 |
| NLP + GBM | 1.0000 | 0.9968 | 0.9852 | 0.9707 |
| **Proposed pipeline** | | | | |
| GloVe + CNN | 0.9998 | 0.9976 | 0.9937 | 0.9811 |

**Table S2-6. Training and testing AUC of G00-G99 (Diseases of the nervous system) in real-world situation.**

| | Training set | | Testing set | |
|---|---|---|---|---|
| | AUC | F-measure | AUC | F-measure |
| **Traditional pipeline** | | | | |
| NLP + SVM (linear) | 0.9967 | 0.9697 | 0.9573 | 0.8629 |
| NLP + SVM (polynomial) | 0.9175 | 0.4967 | 0.8590 | 0.3819 |
| NLP + SVM (radial basis) | 0.9730 | 0.7843 | 0.9524 | 0.7253 |
| NLP + SVM (sigmoid) | 0.9724 | 0.7844 | 0.9519 | 0.7247 |
| NLP + RF | 1.0000 | 0.9961 | 0.9660 | 0.8929 |
| NLP + GBM | 0.9997 | 0.9867 | 0.9626 | 0.8839 |

| | | | | |
|---|---|---|---|---|
| **Proposed pipeline** | | | | |
| GloVe + CNN | 0.9983 | 0.9889 | 0.9771 | 0.9297 |

**Table S2-7. Training and testing AUC of H00-H59 (Diseases of the eye and adnexa) in real-world situation.**

| | **Training set** | | **Testing set** | |
|---|---|---|---|---|
| | **AUC** | **F-measure** | **AUC** | **F-measure** |
| **Traditional pipeline** | | | | |
| NLP + SVM (linear) | 0.9992 | 0.9904 | 0.9784 | 0.9295 |
| NLP + SVM (polynomial) | 0.9882 | 0.8660 | 0.9486 | 0.7917 |
| NLP + SVM (radial basis) | 0.9527 | 0.8846 | 0.9556 | 0.8805 |
| NLP + SVM (sigmoid) | 0.9523 | 0.8839 | 0.9555 | 0.8801 |
| NLP + RF | 1.0000 | 0.9982 | 0.9876 | 0.9483 |
| NLP + GBM | 1.0000 | 0.9953 | 0.9834 | 0.9472 |
| **Proposed pipeline** | | | | |
| GloVe + CNN | 0.9998 | 0.9982 | 0.9868 | 0.9547 |

**Table S2-8. Training and testing AUC of H60-H95 (Diseases of the ear and mastoid process) in real-world situation.**

| | **Training set** | | **Testing set** | |
|---|---|---|---|---|
| | **AUC** | **F-measure** | **AUC** | **F-measure** |
| **Traditional pipeline** | | | | |
| NLP + SVM (linear) | 0.9998 | 0.9922 | 0.9854 | 0.9008 |
| NLP + SVM (polynomial) | 0.9707 | 0.6185 | 0.9100 | 0.4547 |
| NLP + SVM (radial basis) | 0.9827 | 0.8880 | 0.9807 | 0.8420 |
| NLP + SVM (sigmoid) | 0.9833 | 0.8867 | 0.9811 | 0.8411 |
| NLP + RF | 1.0000 | 0.9935 | 0.9781 | 0.9580 |
| NLP + GBM | 1.0000 | 0.9909 | 0.9799 | 0.9320 |
| **Proposed pipeline** | | | | |
| GloVe + CNN | 0.9999 | 0.9994 | 0.9870 | 0.9574 |

**Table S2-9. Training and testing AUC of I00-I99 (Diseases of the circulatory system) in real-world situation.**

| | **Training set** | | **Testing set** | |
|---|---|---|---|---|
| | **AUC** | **F-measure** | **AUC** | **F-measure** |
| **Traditional pipeline** | | | | |
| NLP + SVM (linear) | 0.9956 | 0.9833 | 0.9830 | 0.9565 |
| NLP + SVM (polynomial) | 0.9111 | 0.7378 | 0.9054 | 0.7377 |
| NLP + SVM (radial basis) | 0.9590 | 0.8772 | 0.9637 | 0.8845 |
| NLP + SVM (sigmoid) | 0.9513 | 0.8552 | 0.9559 | 0.8580 |
| NLP + RF | 0.9996 | 0.9946 | 0.9854 | 0.9660 |

| | | | | |
|---|---|---|---|---|
| NLP + GBM | 0.9997 | 0.9919 | 0.9850 | 0.9660 |
| **Proposed pipeline** | | | | |
| GloVe + CNN | 0.9957 | 0.9760 | 0.9878 | 0.9639 |

**Table S2-10. Training and testing AUC of J00-J99 (Diseases of the respiratory system) in real-world situation.**

| | Training set | | Testing set | |
|---|---|---|---|---|
| | **AUC** | **F-measure** | **AUC** | **F-measure** |
| **Traditional pipeline** | | | | |
| NLP + SVM (linear) | 0.9977 | 0.9849 | 0.9800 | 0.9365 |
| NLP + SVM (polynomial) | 0.9150 | 0.5799 | 0.8963 | 0.5808 |
| NLP + SVM (radial basis) | 0.9676 | 0.8120 | 0.9632 | 0.8087 |
| NLP + SVM (sigmoid) | 0.9675 | 0.8118 | 0.9631 | 0.8084 |
| NLP + RF | 1.0000 | 0.9973 | 0.9870 | 0.9586 |
| NLP + GBM | 0.9999 | 0.9935 | 0.9846 | 0.9588 |
| **Proposed pipeline** | | | | |
| GloVe + CNN | 0.9988 | 0.9891 | 0.9869 | 0.9568 |

**Table S2-11. Training and testing AUC of K00-K95 (Diseases of the digestive system) in real-world situation.**

| | Training set | | Testing set | |
|---|---|---|---|---|
| | **AUC** | **F-measure** | **AUC** | **F-measure** |
| **Traditional pipeline** | | | | |
| NLP + SVM (linear) | 0.9938 | 0.9723 | 0.9684 | 0.9182 |
| NLP + SVM (polynomial) | 0.9087 | 0.6485 | 0.8693 | 0.6147 |
| NLP + SVM (radial basis) | 0.9497 | 0.8094 | 0.9329 | 0.7742 |
| NLP + SVM (sigmoid) | 0.9496 | 0.8094 | 0.9328 | 0.7741 |
| NLP + RF | 0.9999 | 0.9946 | 0.9790 | 0.9375 |
| NLP + GBM | 0.9998 | 0.9942 | 0.9808 | 0.9373 |
| **Proposed pipeline** | | | | |
| GloVe + CNN | 0.9960 | 0.9711 | 0.9827 | 0.9389 |

**Table S2-12. Training and testing AUC of L00-L99 (Diseases of the skin and subcutaneous tissue) in real-world situation.**

| | Training set | | Testing set | |
|---|---|---|---|---|
| | **AUC** | **F-measure** | **AUC** | **F-measure** |
| **Traditional pipeline** | | | | |
| NLP + SVM (linear) | 0.9958 | 0.9599 | 0.9538 | 0.8049 |
| NLP + SVM (polynomial) | 0.9456 | 0.5716 | 0.8621 | 0.4318 |
| NLP + SVM (radial basis) | 0.9683 | 0.7583 | 0.9504 | 0.7391 |
| NLP + SVM (sigmoid) | 0.9677 | 0.7582 | 0.9498 | 0.7377 |

| | | | | |
|---|---|---|---|---|
| NLP + RF | 1.0000 | 0.9913 | 0.9444 | 0.8604 |
| NLP + GBM | 0.9996 | 0.9825 | 0.9470 | 0.8509 |
| **Proposed pipeline** | | | | |
| GloVe + CNN | 0.9985 | 0.9914 | 0.9682 | 0.9263 |

**Table S2-13. Training and testing AUC of M00-M99 (Diseases of the musculoskeletal system and connective tissue) in real-world situation.**

| | Training set | | Testing set | |
|---|---|---|---|---|
| | **AUC** | **F-measure** | **AUC** | **F-measure** |
| **Traditional pipeline** | | | | |
| NLP + SVM (linear) | 0.9932 | 0.9620 | 0.9653 | 0.9094 |
| NLP + SVM (polynomial) | 0.9494 | 0.7045 | 0.9010 | 0.5915 |
| NLP + SVM (radial basis) | 0.9548 | 0.7988 | 0.9383 | 0.7734 |
| NLP + SVM (sigmoid) | 0.9547 | 0.7989 | 0.9381 | 0.7734 |
| NLP + RF | 0.9990 | 0.9891 | 0.9711 | 0.9257 |
| NLP + GBM | 0.9997 | 0.9884 | 0.9742 | 0.9197 |
| **Proposed pipeline** | | | | |
| GloVe + CNN | 0.9948 | 0.9713 | 0.9769 | 0.9345 |

**Table S2-14. Training and testing AUC of N00-N99 (Diseases of the genitourinary system) in real-world situation.**

| | Training set | | Testing set | |
|---|---|---|---|---|
| | **AUC** | **F-measure** | **AUC** | **F-measure** |
| **Traditional pipeline** | | | | |
| NLP + SVM (linear) | 0.9935 | 0.9713 | 0.9660 | 0.9016 |
| NLP + SVM (polynomial) | 0.9288 | 0.6838 | 0.9217 | 0.7008 |
| NLP + SVM (radial basis) | 0.9594 | 0.7998 | 0.9635 | 0.8415 |
| NLP + SVM (sigmoid) | 0.9593 | 0.7997 | 0.9634 | 0.8415 |
| NLP + RF | 0.9999 | 0.9953 | 0.9733 | 0.9246 |
| NLP + GBM | 0.9997 | 0.9918 | 0.9726 | 0.9219 |
| **Proposed pipeline** | | | | |
| GloVe + CNN | 0.9962 | 0.9756 | 0.9842 | 0.9492 |

**Table S2-15. Training and testing AUC of O00-O9A (Pregnancy, childbirth and the puerperium) in real-world situation.**

| | Training set | | Testing set | |
|---|---|---|---|---|
| | **AUC** | **F-measure** | **AUC** | **F-measure** |
| **Traditional pipeline** | | | | |
| NLP + SVM (linear) | 0.9999 | 0.9980 | 0.9971 | 0.9890 |
| NLP + SVM (polynomial) | 0.9988 | 0.9600 | 0.9929 | 0.9046 |
| NLP + SVM (radial basis) | 0.9978 | 0.9720 | 0.9945 | 0.9692 |

| | | | | |
|---|---|---|---|---|
| NLP + SVM (sigmoid) | 0.9978 | 0.9720 | 0.9944 | 0.9689 |
| NLP + RF | 1.0000 | 0.9982 | 0.9960 | 0.9875 |
| NLP + GBM | 0.9999 | 0.9980 | 0.9988 | 0.9887 |
| **Proposed pipeline** | | | | |
| GloVe + CNN | 0.9999 | 0.9996 | 0.9980 | 0.9919 |

**Table S2-16. Training and testing AUC of P00-P96 (Certain conditions originating in the perinatal period) in real-world situation.**

| | **Training set** | | **Testing set** | |
|---|---|---|---|---|
| | **AUC** | **F-measure** | **AUC** | **F-measure** |
| **Traditional pipeline** | | | | |
| NLP + SVM (linear) | 0.9992 | 0.9810 | 0.9930 | 0.9471 |
| NLP + SVM (polynomial) | 0.9931 | 0.8208 | 0.9930 | 0.7821 |
| NLP + SVM (radial basis) | 0.9936 | 0.8907 | 0.9952 | 0.9118 |
| NLP + SVM (sigmoid) | 0.9935 | 0.8907 | 0.9952 | 0.9118 |
| NLP + RF | 0.9998 | 0.9812 | 0.9879 | 0.9418 |
| NLP + GBM | 0.9996 | 0.9778 | 0.9800 | 0.9471 |
| **Proposed pipeline** | | | | |
| GloVe + CNN | 0.9998 | 0.9989 | 0.9978 | 0.9758 |

**Table S2-17. Training and testing AUC of Q00-Q99 (Congenital malformations, deformations and chromosomal abnormalities) in real-world situation.**

| | **Training set** | | **Testing set** | |
|---|---|---|---|---|
| | **AUC** | **F-measure** | **AUC** | **F-measure** |
| **Traditional pipeline** | | | | |
| NLP + SVM (linear) | 0.9934 | 0.9230 | 0.9143 | 0.7376 |
| NLP + SVM (polynomial) | 0.8983 | 0.3187 | 0.7808 | 0.1655 |
| NLP + SVM (radial basis) | 0.9729 | 0.7643 | 0.9052 | 0.6208 |
| NLP + SVM (sigmoid) | 0.9713 | 0.7539 | 0.9076 | 0.6202 |
| NLP + RF | 0.9999 | 0.9707 | 0.9120 | 0.7509 |
| NLP + GBM | 0.9988 | 0.9353 | 0.8956 | 0.6882 |
| **Proposed pipeline** | | | | |
| GloVe + CNN | 0.9993 | 0.9955 | 0.9055 | 0.7819 |

**Table S2-18. Training and testing AUC of R00-R99 (Symptoms, signs and abnormal clinical and laboratory findings, not elsewhere classified) in real-world situation.**

| | **Training set** | | **Testing set** | |
|---|---|---|---|---|
| | **AUC** | **F-measure** | **AUC** | **F-measure** |
| **Traditional pipeline** | | | | |
| NLP + SVM (linear) | 0.9809 | 0.8829 | 0.8898 | 0.7365 |
| NLP + SVM (polynomial) | 0.8693 | 0.4650 | 0.8193 | 0.4042 |

| | | | | |
|---|---|---|---|---|
| NLP + SVM (radial basis) | 0.9442 | 0.6978 | 0.9099 | 0.6404 |
| NLP + SVM (sigmoid) | 0.9433 | 0.6962 | 0.9090 | 0.6390 |
| NLP + RF | 0.9967 | 0.9759 | 0.9091 | 0.7372 |
| NLP + GBM | 0.9993 | 0.9805 | 0.9172 | 0.7600 |
| **Proposed pipeline** | | | | |
| GloVe + CNN | 0.9872 | 0.9573 | 0.9399 | 0.8726 |

**Table S2-19. Training and testing AUC of S00-T88 (Injury, poisoning and certain other consequences of external causes) in real-world situation.**

| | Training set | | Testing set | |
|---|---|---|---|---|
| | **AUC** | **F-measure** | **AUC** | **F-measure** |
| **Traditional pipeline** | | | | |
| NLP + SVM (linear) | 0.9840 | 0.9206 | 0.8861 | 0.7366 |
| NLP + SVM (polynomial) | 0.9301 | 0.6424 | 0.8463 | 0.5729 |
| NLP + SVM (radial basis) | 0.9094 | 0.6930 | 0.8617 | 0.6816 |
| NLP + SVM (sigmoid) | 0.9092 | 0.6930 | 0.8614 | 0.6815 |
| NLP + RF | 0.9993 | 0.9787 | 0.8481 | 0.7448 |
| NLP + GBM | 0.9995 | 0.9784 | 0.8840 | 0.7481 |
| **Proposed pipeline** | | | | |
| GloVe + CNN | 0.9941 | 0.9689 | 0.9271 | 0.8386 |

**Table S2-20. Training and testing AUC of V00-Y99 (External causes of morbidity) in real-world situation.**

| | Training set | | Testing set | |
|---|---|---|---|---|
| | **AUC** | **F-measure** | **AUC** | **F-measure** |
| **Traditional pipeline** | | | | |
| NLP + SVM (linear) | 0.9699 | 0.3938 | 0.7448 | 0.0722 |
| NLP + SVM (polynomial) | 0.6930 | 0.0723 | 0.6054 | 0.0178 |
| NLP + SVM (radial basis) | 0.9379 | 0.4137 | 0.7386 | 0.0468 |
| NLP + SVM (sigmoid) | 0.8735 | 0.3088 | 0.5977 | 0.0248 |
| NLP + RF | 0.9997 | 0.9313 | 0.6939 | 0.0246 |
| NLP + GBM | 0.9995 | 0.9269 | 0.6577 | 0.0721 |
| **Proposed pipeline** | | | | |
| GloVe + CNN | 0.9986 | 0.9878 | 0.8086 | 0.3257 |

**Table S2-21. Training and testing AUC of Z00-Z99 (Factors influencing health status and contact with health services) in real-world situation.**

| | Training set | | Testing set | |
|---|---|---|---|---|
| | **AUC** | **F-measure** | **AUC** | **F-measure** |
| **Traditional pipeline** | | | | |
| NLP + SVM (linear) | 0.9562 | 0.8711 | 0.8398 | 0.7094 |

| | | | | |
|---|---|---|---|---|
| NLP + SVM (polynomial) | 0.7582 | 0.5546 | 0.7435 | 0.5535 |
| NLP + SVM (radial basis) | 0.8631 | 0.6789 | 0.8223 | 0.6394 |
| NLP + SVM (sigmoid) | 0.8630 | 0.6787 | 0.8222 | 0.6392 |
| NLP + RF | 0.9981 | 0.9754 | 0.8790 | 0.6932 |
| NLP + GBM | 0.9961 | 0.9634 | 0.8536 | 0.7056 |
| **Proposed pipeline** | | | | |
| GloVe + CNN | 0.9623 | 0.8976 | 0.8947 | 0.7833 |
